# Synergistic Effects of Nanoparticles and Surface Anchoring on Fine-Tuning the Photonic Bandgap in Blue Phase Liquid Crystals

**DOI:** 10.1021/acsnano.6c01127

**Published:** 2026-06-08

**Authors:** Kamil Orzechowski, Martyna Wasiluk, Weronika Milewska, Natalia Kowalska, Yi-Te Chuang, Jia-Yu Cao, Chun-Ta Wang, Aleksandra Neumann, Olga Strzeżysz, Anna Kozanecka-Szmigiel, Jolanta Konieczkowska, Ewa Schab-Balcerzak, Wiktor Lewandowski, Tomasz R. Woliński

**Affiliations:** † Faculty of Physics, 232858 Warsaw University of Technology, Koszykowa 75 Str., Warsaw 00-662, Poland; ‡ Faculty of Chemistry, 49605 University of Warsaw, Pasteura 1 Str., Warsaw 02-093, Poland; § Department of Photonics, 34874 National Sun Yat-sen University, No. 70 Lien-hai Rd., Kaohsiung 80424, Taiwan; ∥ Institute of Chemistry, Military University of Technology, Kaliskiego 2 Str., Warsaw 00-908, Poland; ⊥ Centre of Polymer and Carbon Materials, 111480 Polish Academy of Sciences, M. Curie-Sklodowska 34 Str., Zabrze 41-819, Poland; # Institute of Chemistry, University of Silesia, Szkolna 9 Str., Katowice 40-007, Poland

**Keywords:** liquid crystal, blue phase, photonic bandgap, functionalized gold nanoparticles, liquid-crystal-like ligand shell, alignment layers, thermal stability

## Abstract

Precise control over the photonic bandgap in Blue Phase Liquid Crystals (BPLCs) remains challenging due to the inherent limitations of existing tuning methods. Here, we present a 2-fold approach that synergistically combines internal and external effectors to enable controlled, fine modulation of the photonic bandgap across a wide spectral range of 200 nm. Internally, nanoparticles (NPs) embedded within the BPLC lattice enhance the thermal stability of the blue phase and reduce the cubic unit cell size, thereby shifting the reflection bandgap toward shorter wavelengths. Externally, the chemical structure of homogeneous alignment layers (ALs) affects the spectral position of the Bragg reflection. By systematically varying four ALs and three NP doping levels (0, 0.5, and 2 wt %), a cooperative influence of both effectors on spectral tuning is observed. These interactions are qualitatively explained by contact-angle measurements and chemical interactions at the LC-AL and LC-NP interfaces. Kossel diagram analysis, together with a factor based on the total tuning range and associated statistical descriptors, is used to confirm and quantify Bragg wavelength shifts. The results demonstrate that combined internal and external control provides an effective strategy for adjusting the optical response and thermal behavior of BPLCs, supporting their application in photonic devices.

## Graphical abstract

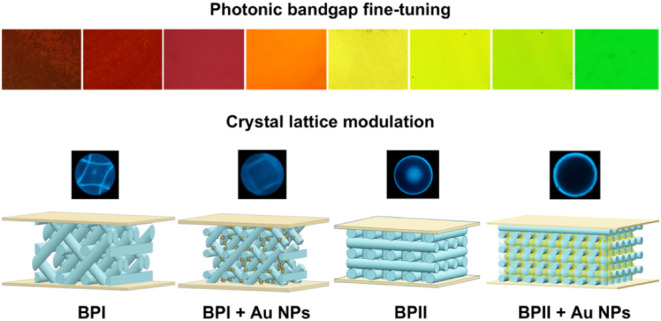

## Introduction

Since Yablonovitch introduced the concept of photonic crystals—materials that provide access to a photonic bandgap analogous to the electronic bandgap of semiconductors—there has been significant progress in the evolution of technologies revolving around light flow control.^1^ This advancement enabled the development of versatile photonic materials that allow for the precise engineering of light propagation, enhancing or suppressing emission in the spectral range of interest and acting on both frequency and time domains.^2–5^ If these properties are set in the visible, and the material is responsive to external stimuli, systems with a facile naked-eye readout to external stress can be developed. Such structures are particularly valuable for optical quantum and fiber-optic communications, intelligent and miniaturized optical multisensors, secure anticounterfeiting systems, and other technologies.^6–11^

Despite these rapid advancements, it remains challenging to develop materials that enable precise tuning of their initial photonic bandgap with high precision over a wide spectral range, required for advanced optical communication and sensor systems. The lack of such precise control stems from the fundamental limitations of material fabrication techniques, which makes the challenge both captivating from the intellectual and application-wise perspectives.

From the materials’ point of view, several systems enable light propagation in the visible spectrum. These include cholesteric^12^ liquid crystals (LCs), block copolymers,^13^ colloidal inks assisted by hydrogen bonds and processed using three-dimensional (3D) printing technology,^14^ as well as cellulose^15^ and halide perovskite^16,17^ nanocrystals forming a long-range chiral nematic organization. However, among them, blue-phase liquid crystals^18^ (BPLCs) seem particularly appealing for the progression of advanced tunable and multiresponsive devices due to their structurally complex LC phase, which exhibit electro-, magneto-, and acousto-optic coefficients with an order of magnitude greater than any other material.^19^ Originally dedicated to advanced and high-speed display systems,^20^ BPLCs have recently been applied to a wide range of photonic technologies.^21–27^

The applicability of BPLCs stems from their well-defined 3D chiral structure. Chiral systems are known to strongly reflect light whose wavelength matches their helical pitch, but what makes BPLCs exceptional is their ability to form chiral entities along multiple axes. They exhibit two distinct thermosensitive mesophases, BPI and BPII, within a narrow temperature range (∼0.1–5.0 °C). In both BP phases, the cubic unit cell is constructed from double-twist cylinders (DTCs)^28^—self-assembled molecular architectures in which the director twists in two perpendicular directions, aligning with either a full or half pitch for BPI and BPII, respectively. In BPI, the DTCs weave into a body-centered cubic lattice (space group *O* ^8^, *I*4_1_32), whereas in BPII they adopt a simple cubic arrangement (space group *O* ^2^, *P*4_2_32). The differences between the two BP phases arise from how these DTCs pack and from the organization of the resulting disclination network.

Owing to lattice spacings on the order of several hundred nanometers, BPLCs strongly interact with visible light. This interaction gives rise to vivid structural colors through a 3D photonic bandgap, manifested as Bragg reflections from specific cubic lattice planes.^29^ For normal incidence of a light beam on the BPLC structure, the reflected wavelength λ_B_ satisfies the Bragg condition:

1 $$\lambda_{B}=\frac{2ña}{\sqrt{h^{2}+k^{2}+l^{2}}}$$

Here, ñ and *a* represent the average refractive index and lattice constant of BPLCs, respectively, while (*hkl*) are the Miller indices of the reflecting planes.

Within the realm of BPLCs, controlling the photonic properties has been shown to be challenging, although it has been extensively undertaken recently. A particular cause of this interest was propelled by the discovery that introducing optically active additives, known as chiral dopants, to nematic LCs^30^ can affect the helical pitch and lattice constants, thereby shifting Bragg reflections. The shifts in Bragg reflections in both chiral nematic (N*) and BP phases manifest differently due to their differing dimensional structures, with higher chiral dopant concentrations reducing lattice constants and changing lattice orientation in BPs,^31^ as presented in a subsequent section of the text.

To date, many known approaches primarily allow for the dynamic tuning of the Bragg wavelength using electric^32,33^ and optical^34^ fields, heating,^35^ or mechanical stress^36^ (Figure 1a). Often, polymer stabilization^20^ of BPLCs is required for efficient operation. However, these methods provide only metastable states with a specific color reflection in BPs. Selective light reflection in BPLCs is also influenced by boundary conditions such as surface pinning effects, confinement geometries, and chemical functionalization of substrates.^37–42^ The latter relies on using particular alignment layers (ALs) that provide a homeotropic or homogeneous orientation of LC molecules on substrates, thereby affecting nucleation during BP crystal growth, reflection color, and domain morphology.^31,38,43,44^ Moreover, the homogeneity and lattice orientation of BP domains are significantly influenced by the specific distribution of ALs on the substrates, as well as the alignment technique (e.g., rubbing or photoalignment),^45–50^ which is crucial for practical LC-based devices.

**Figure 1:**
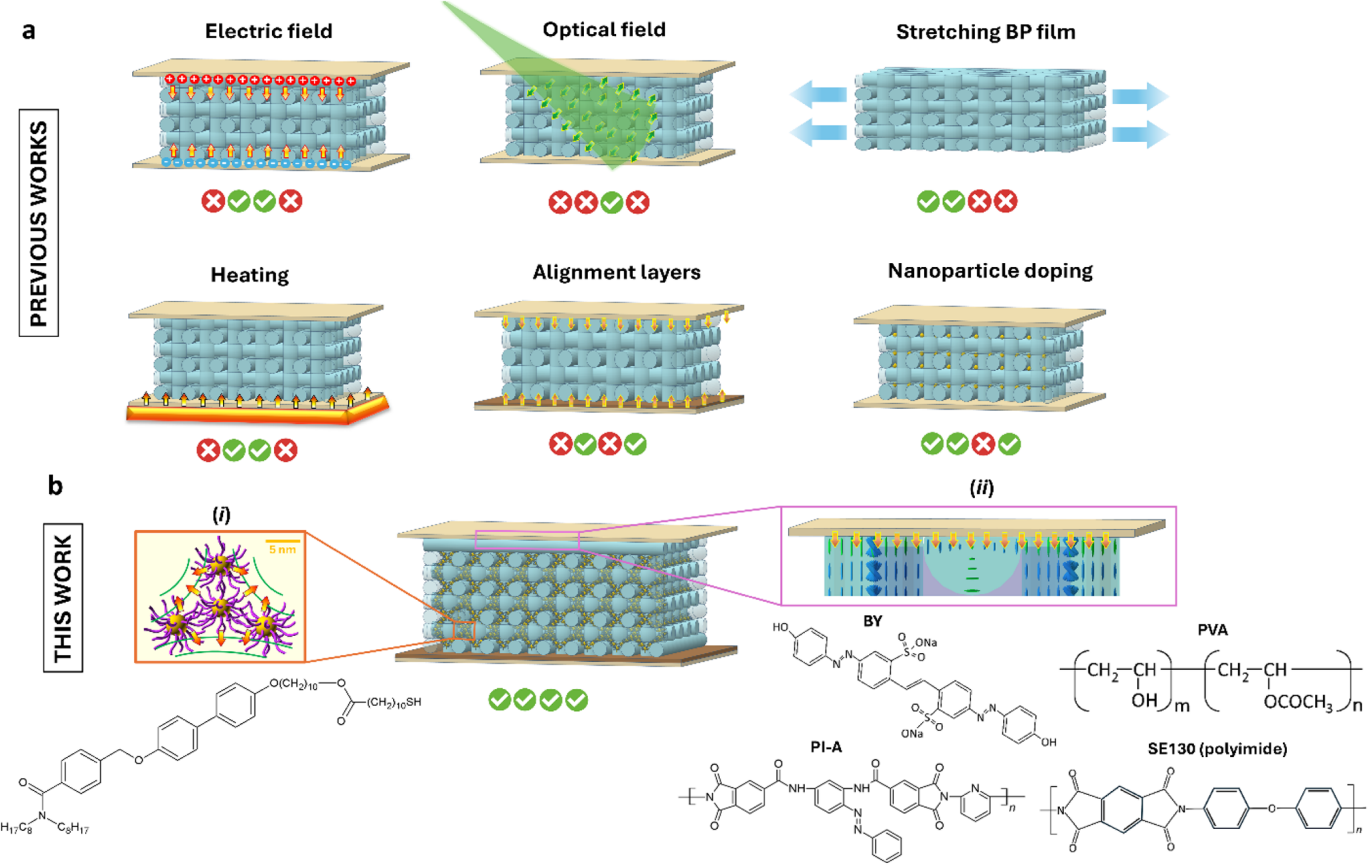
Photonic bandgap tuning—traditional vs the innovations introduced here. (a) Traditional methods influencing the lattice size in BPLCs. Small circles (red or green) represent benchmarks; in the order of their appearance, they represent enhancing BP stability, fine-tuning the photonic bandgap (<10 nm), tuning over a wide spectral range (>150 nm), and preserving the initial lattice symmetry within a specific BP phase. (b) The 2-fold approach introduced here encompasses external and internal effectors. (i) Internal (on the left). The internal approach relies on nanoparticles, which cause a lowering of the cholesteric pitch of BP; arrows represent the idea of strong interactions between BPLC molecules from DTCs (green lines) and ligands (violet curved lines), causing the DTC structures to come closer and a reduction in the pitch length; surface ligands of nanoparticles are shown below; one of these ligands exhibits a liquid-crystal-like structure which will be important in the context of nanoparticle role; (ii) external. On the right, the external approach is exerted through the interaction between BPLC molecules near the boundary and the alignment layers, influenced by surface anchoring energy.

Besides the above-mentioned methods, doping BPLCs with nanoparticles (NPs) enhances thermal stability and shifts Bragg reflections, typically toward longer wavelengths at higher NP concentrations. The shift’s magnitude depends on the dielectric properties, dimensions, and shape of the used NPs.^51–55^ This phenomenon can be interpreted as the energetic penalty associated with disclinations driving the DTC structures apart, thereby enlarging the BP unit cell beyond what the equilibrium chiral twist would naturally dictate.^54^ However, specific chemical functionalization of the NP surface enables the control of the Bragg wavelength shift toward shorter wavelengths with increasing NP concentration in BPLC.^56^ This results from the interaction, e.g., between designed NP ligands with LC-like properties and LC molecules in DTCs, shortening the DTC helical pitch and reducing the BP unit-cell size. NP doping of BPLCs can bring promising advancements with enhanced properties. Their ability to alter photonic properties and simultaneously boost the thermal characteristics of BPs may spark considerable interest in the design of tunable 3D microstructures. Yet, a significant challenge remains in achieving a specific Bragg reflection from a wide spectral range in thermally stable BPs, ensuring long-term chemical stability between the selected BPLC and nano dopants.

Here, we develop the concept of merging *external* and *internal effectors* to ensure extensive tunability and exceptional thermal stability of BPLCs (Figure 1b), showcasing diverse techniques for modulating the Bragg wavelength in BPs and their synergistic potential. In particular, we prepared a series of BPLC composite samples in which the BPLC is doped with varying concentrations of chiral and Au NP dopants. These NPs act as *internal effectors* that ensure the blue-shifting of the Bragg wavelength, with the magnitude of this effect being dependent on the NP concentrations. Additionally, homogeneous ALs with tailored surface anchoring energies serve as *external effectors*, extending the spectral modulation range. Our approach preserves the initial lattice orientation and crystallographic symmetry of the soft 3D nanostructure within a specific BP. Overall, these findings demonstrate the effect of NP doping on BPLC properties and provide guidance for tailoring them to meet the requirements of photonic applications.

## Results and Discussion

### Controlling the Photonic Properties Using Traditional Effectors

Three BPLC mixtures were prepared for this study, designated as BPLC #1, BPLC #2, and BPLC #3, containing 11.1, 14.2, and 17.4 wt % of chiral dopants in the nematic mixture, respectively (Tables S1 and S2, Supporting Information). Details regarding the preparation and investigation of the BPLC samples are provided in the Experimental section. Although most of the work presented in this article will be based on BPLC #2, the other mixtures will highlight the need for developing advanced control over BPs. We begin the analysis with samples placed in LC cells with ALs having a relatively high anchoring energy, rubbed polyimides (W ≈ 10^–3^ J·m^–2^, SE130, Nissan Chemical Industries, Ltd.).^57^ The initial tests described below were conducted to explore the common methods for BPLC control.

First, we note that testing several BPLC materials with different chiral dopant types and concentrations is necessary to determine a clear relationship between the Bragg wavelength and the chiral dopant concentration in BPs.^31^ This relationship is straightforward in the N* phase of the mixtures—the increasing amount of the chiral dopant causes the decrease of the helical pitch length. This pitch is 410, 350, and 280 nm for BPLCs #1, #2, and #3, respectively (Figure S1, Supporting Information), resulting in a sequential change in reflection color from red (670 nm) to green (530 nm) to blue (445 nm). However, this monotonic and foreseeable change is not observed in the selective reflection of BP phases (Figure 2a). The reflectance of BPII phases changed from blue (488 nm) to yellow (595 nm) to blue (478 nm) with growing amounts of the chiral dopant. This nonmonotonic change often results from rotating the facet normal to the substrate from (110)_BPII_ to (100)_BPII_ (Figure S2, Supporting Information), and indeed, the Kossel diagrams of these films confirm this rotation (Figure 2a, insets). Beyond foreseeable variation, another crucial aspect in designing BPLCs is achieving monodomain structures over a large sample area (at least several mm^2^). This is particularly demanding in the BPI case (Supporting Note 1). Although large domains with different reflections are typically visible in BPI, achieving a monodomain texture for the BPLC with a specific helical pitch is possible, as demonstrated for BPLC #3.

**Figure 2:**
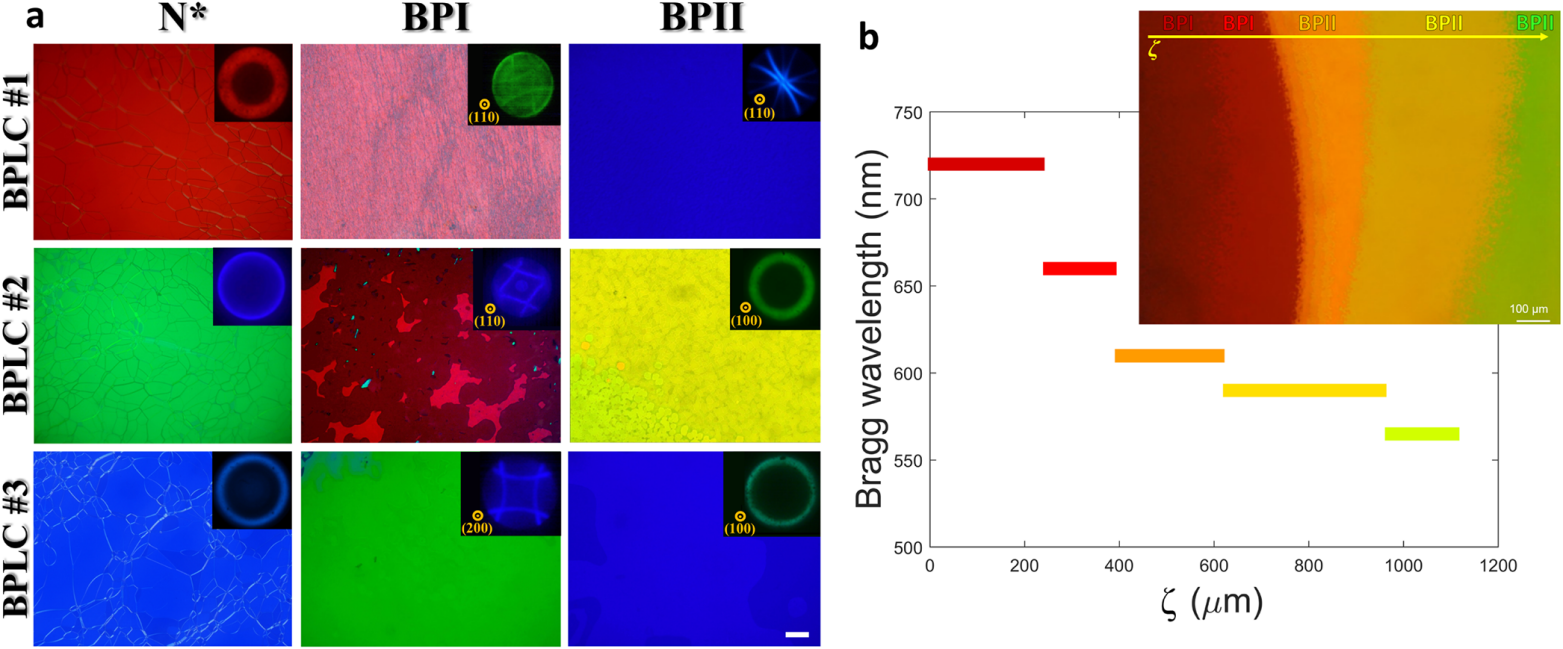
Photonic bandgap tuning using chiral dopants and gradient temperature. (a) Polarized optical microscopy (POM) images show the LC textures obtained from 12 μm-thick LC samples with homogeneous alignment layers that possess relatively high anchoring energy, and insets display the recorded Kossel diagrams with specified Miller indices for the subsequent LC phases (BPII, BPI, N*) during cooling. The BPLCs contain chiral dopants of the following concentrations: 11.1 wt % (BPLC #1), 14.2 wt % (BPLC #2), and 17.4 wt % (BPLC #3). Kossel patterns were recorded using converging monochromatic light at wavelengths of 488, 560, or 650 nm. The white scale bar corresponds to 200 μm. (b) A graph of the Bragg wavelengths measured along the yellow arrow in the POM image (horizontal position in the POM image, ζ), which shows colorful stripes resulting from the selective reflection of monochromatic light, ranging from red to green, achieved through a temperature gradient.

Second, we explored the possibility of obtaining a continuous variation of the Bragg peak using a more advanced approach that relies on a temperature gradient centered around the BPI-BPII phase transition. We used the BPLC #2 mixture in this experiment, which exhibited the broadest spectral range of Bragg reflection across this transition among all tested mixtures (Figure S3, Supporting Information). To generate the gradient, one part of the sample was placed on a stage set to a temperature within the BPI range (low-temperature stage), while the other part was positioned on a stage set to a BPII temperature (high-temperature stage), following a setup similar to that previously used by Chen et al. to grow large crystals within a single BP phase,^58^ though not across a phase boundary. We observed a spatial distribution of monochromatic light reflections, ranging from red to green (Figure 2b). However, the variation was not continuous; instead, narrow colored stripes formed with distinct boundaries (Supporting Note 2).

Lastly, we emphasize that surface anchoring energy predominates when the sample thickness is minimal (on the order of a few micrometers) and becomes negligible for greater thicknesses (dozens of micrometers and beyond). By applying varying boundary forces to the LC molecules along one direction in the sample, spatial modulation of light reflections in BPLC can be achieved (Supporting Note 3).

Overall, the experiments as mentioned above suggest that standard methods for controlling BPLCs fail to produce a material with a continuous, perfectly controlled change in Bragg wavelength. Consequently, it is impossible to overcome the current limits of BPLCs in terms of the broadness and precision of the continuous change in Bragg reflection, as well as the limited thermal stability of the obtained reflecting regions. Therefore, in the following part of this work, we focus on the external-internal approach for controlling the photonic properties in BPLCs:

(i) *external effector*—the application of ALs with varying anchoring energies in flat cells filled with a single BPLC material (BPLC #2) and
(ii) *internal effector*—by incorporating gold nanoparticles (Au NPs) at various concentrations.

### Designing External Effectors (Alignment Layers) for Photonic Control

Regarding the *external effectors*, four types of ALs promoting homogeneous LC alignment were applied to the samples, chosen to provide varied surface anchoring energies, which may potentially influence the helical twist in BPLC. The ALs utilized include brilliant yellow azo-dye (BY, Sigma-Aldrich), poly­(vinyl alcohol) (PVA, Sigma-Aldrich), polyimide (SE130, Nissan Chemical Industries, Ltd.), and a custom-synthesized azo poly­(amide imide) material (PI-A). The latter, developed to achieve intermediate surface anchoring energy, had not been previously employed in any BPLC study. Detailed characteristics of the PI-A material are provided in the Experimental section. Comprehensive information regarding all ALs used in this study, along with protocols for their application on substrates, is provided in theExperimental Section and Table S4 (Supporting Information). Notably, the set of ALs used in this study allows us to explore a broad range of molecular interactions with BPLCs. Based on their molecular architectures, BY is expected to exhibit the lowest azimuthal anchoring energy due to its highest polarity and potential for π–π interactions. On the other hand, SE130 likely provides the strongest interactions owing to its pronounced aromatic backbone, which facilitates π–π stacking and dipolar interactions. PVA demonstrates an intermediate level of interaction, primarily through its affinity for the alkyl portions of BPLCs, while not ensuring π–π stacking due to its nonaromatic nature. Lastly, the custom PI-A supports π–π interactions but is less planar and more polar than SE130, placing its interaction strength between those of BY and SE130 ALs.

To experimentally assess differences in surface interactions among the four ALs, we measured the static contact angle (α) of deionized water and the 1912 nematic LC, which serves as the base compound of the BPLC mixture used in subsequent optical studies. For each substrate, measurements were taken on both sides of the droplet, with ten replicates per side, to determine the average contact angle and its standard deviation (see the Experimental section and Table S5, Supporting Information). The obtained water contact angles were 5.4 ± 0.1°, 40.2 ± 0.3°, 68.1 ± 0.2°, and 87.6 ± 0.1° for BY, PVA, PI-A, and SE130, respectively (Figure 3a). This represents a shift from strongly hydrophilic to moderately hydrophilic, and finally to nearly hydrophobic materials. Using the Young-Dupré relation (eq 2), this trend translates into a monotonic decrease in the work of adhesion (*W*_*adh*_) from BY to SE130, indicating progressively weaker polar interactions.

**Figure 3:**
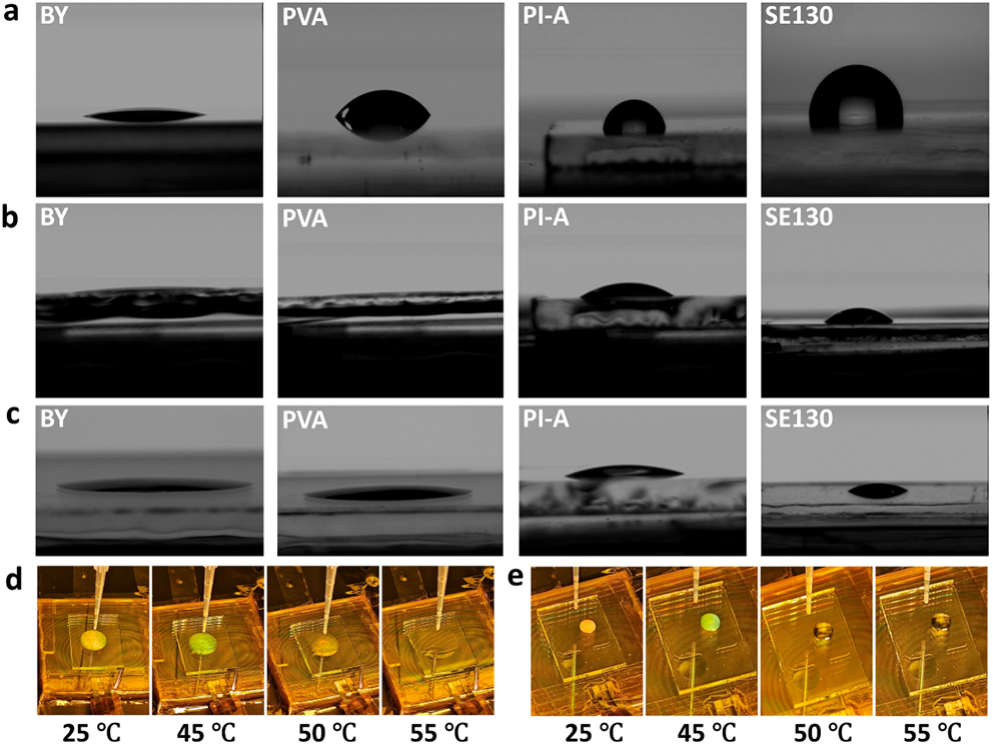
Surface anchoring energy analysis. Images of a drop of (a) deionized water, (b) the 1912 nematic LC, and (c) BPLC #2 were recorded for the contact angle measurements at 25 °C. Top-view images of a BPLC #2 droplet at different temperatures are presented for (d) BY and (e) SE130 ALs.

2 $$W_{adh}=\gamma \left(1+\cos\alpha \right)$$

where γ = 72 × 10^–3^ J·m^–2^ is the surface tension of water at 25 °C.^59^ Using this value, the calculated work of adhesion for each AL is as follows: BY – 144 × 10^–3^ J·m^–2^, PVA – 127 × 10^–3^ J·m^–2^, PI-A – 99 × 10^–3^ J·m^–2^, and SE130 – 75 × 10^–3^ J·m^–2^. These values confirm that BY provides the strongest polar interaction with the liquid, followed by PVA, PI-A, and SE130.

In liquid crystal systems, the total anchoring energy is described by the Rapini–Papoular model,^60^ which distinguishes between deviations of the LC director in both polar and azimuthal directions. For minor deviations from the easy axis (i.e., a preferred direction for the LC director at the interface), the total anchoring energy can be expressed as

3 $$f_{s}=\frac{1}{2}W_{\theta }\;sin^{2}\left(\theta -\theta_{0}\right)+\frac{1}{2}W_{\phi }\;sin^{2}\left(\theta_{0}\right)\;sin^{2}\left(\phi -\phi_{0}\right)$$

where *W*_θ_ and *W*_ϕ_ are the polar and azimuthal anchoring strength coefficients, respectively, and θ_0_, ϕ_0_ denote the preferred polar and azimuthal angle of the LC director, respectively. For a homogeneous anchoring of the considered ALs (θ_0_ ≈ *π/2*), defining *ψ = π/2 – θ*, which is the polar angle defined with respect to the substrate surface, simplifies the expression to

4 $$f_{s}=\frac{1}{2}W_{\theta }\;sin^{2}\left(\psi -\psi_{0}\right)+\frac{1}{2}W_{\phi }\;sin^{2}\left(\phi -\phi_{0}\right)$$

In the case of the 1912 nematic LC and the selected BPLC mixture (Figure 3b,c), we observed almost complete spreading at 25 °C for BY and PVA (α ≈ 0°), indicating very strong wetting and high polar anchoring energy. However, contact angles of ∼20° and ∼30° were measured for PI-A and SE130, respectively, for both nematic LC and BPLC. The lower contact angles observed for LCs compared to water are attributed to their polar nature and better compatibility with the ALs. This behavior was further retained at elevated temperatures, with only modest variation in polar anchoring energy (25–70 °C, see Figures S4 and S5, and Table S6 in the Supporting Information, and Supporting Note 4)—Figure 3d,e shows top-view images of BPLC droplets on BY and SE130 substrates, which represent the two extremes of azimuthal anchoring energy. While the droplet on BY spreads completely, also at elevated temperatures, the one on SE130 retains a distinct shape, including in the isotropic phase (e.g., at 55 °C), indicating persistent anchoring constraints.

The qualitative tests confirmed that BY and PVA layers exhibit the strongest polar anchoring, while PI-A and SE130 show progressively weaker polar anchoring. Based on the overall assessment of azimuthal anchoring energy, the ALs can be ranked from weakest to strongest as follows: BY < PVA < PI-A < SE130.

Before proceeding with further analysis, it is essential to note that, in addition to anchoring energy, the molecular composition of ALs plays a crucial role in modulating the chiral twist of BPLCs through interactions with LC molecules that form DTC structures. This influence arises from specific chemical interactions at the LC–AL interface, which can affect the local twist elasticity and pitch. Importantly, this effect is typically considered independent of the natural helical pitch in numerical models of chiral LCs in the BPs, and is primarily responsible for the ordering of BP domains.^61,62^

### Designing Internal Effectors (Nanoparticles) for Photonic Control

As internal effectors, we set out to engineer Au NPs that are (i) small enough to access and eventually localize within the defect network of blue phases, (ii) chemically compatible with the BPLC host so that they disperse uniformly in the bulk yet can be taken up by disclination channels upon crystallization, and (iii) thermally robust across the processing window. To meet these criteria, we obtained 2.3 ± 0.4 nm Au spheres using a modified Brust–Schiffrin approach, initially capped with dodecanethiol (AuNPs@DDT). These seeds were then subjected to a partial ligand exchange with an LC-like thiol, [(4′-{[4-(dioctylcarbamoyl)­phenyl]­methoxy}-[1,1′-biphenyl]-4-yl)­oxy]­undecyl 11-sulfanylundecanoate (Figure S6, Supporting Information). Thermogravimetric analysis indicates that approximately 1/4 of the original DDT molecules are replaced by the LC-like ligand, yielding AuNPs@DDT/LC with a binary organic corona in which the two ligand types contribute comparable mass fractions (Supporting Note 5).

The chemical structure of the nanoparticle shell was crucial to the internal effector feature of NPs. On the one side, the rod-like alkyl/aromatic and amide groups of the LC-like ligand provide van der Waals, π–π and dipolar interaction sites for interfacing with BPLC molecules. On the other side, the LC-like ligands are “diluted” in a DDT matrix, which renders the organic shell relatively compliant: the LC-like chains can form local bundles and adapt to the anisotropic shape of the BPLC defects instead of forming a rigid, densely packed monolayer.^63^ This flexibility of the organic shell is expected to facilitate partial infiltration of BPLC molecules into the corona, lowering the elastic penalty of accommodating NPs in disclination cores, while the relatively thick organic shell maintains colloidal stability and suppresses sintering. To qualitatively test assumptions of the role of the NP-shell, we compared the properties of:

(i) NPs before and after ligand exchange (AuNPs@DDT vs AuNPs@DDT/LC) and
(ii) neat AuNPs@DDT/LC with their composites containing small, controlled BPLC fractions (AuNPs@DDT/LC + 0/5/50 wt % BPLC).

We first verified that ligand exchange neither induces aggregation nor changes the Au core size. TEM analysis revealed that AuNPs@DDT and AuNPs@DDT/LC both consist of nearly spherical particles with indistinguishable size distributions (2.3 ± 0.4 nm vs 2.2 ± 0.4 nm), and no faceted or fused objects are observed within the statistical error (Figure S6, Supporting Information). UV–Vis spectra of AuNPs@DDT and AuNPs@DDT/LC dispersions confirm this picture. The spectra almost perfectly overlap, both displaying a relatively broad LSPR band centered at ∼495 nm (Supporting Note 6). The absence of any red shift or additional low-energy shoulder indicates that interparticle coupling does not increase in solution, i.e., the NPs remain entirely colloidally stable after ligand exchange.

When comparing thin films of AuNPs@DDT and AuNPs@DDT/LC with their dispersions, we observe the expected red shift and broadening of the plasmon band in the solid state, arising from interactions between closely packed NPs and from the higher effective refractive index (Supporting Note 6). Namely, LSPR maxima appear at ∼525 nm for AuNPs@DDT and ∼518 nm for AuNPs@DDT/LC. The slightly smaller red shift in the latter suggests larger average NP separations, consistent with a “looser” packing within the mixed ligand shell. Importantly, no additional low-energy bands characteristic of strongly coupled or sintered particles are detected; thus, dropcasting the material again does not affect the particle structure.

SAXS studies of these materials provide complementary insights into the structural organization. At 30 °C, AuNPs@DDT exhibit a single correlation peak corresponding to a center-to-center distance of ∼3.3 nm. While AuNPs@DDT/LC show two well-defined peaks with periodicities of ∼6.9 and ∼3.4 nm, indicating a more anisotropic short-range organization of the cores within the mixed corona. Upon heating to 140 °C, these peaks merge into a broad signal centered at 3.5–4.4 nm; upon cooling back to 30 °C, the initial two-peak pattern is almost fully restored (Supporting Note 7, Table S7, Supporting Information). This behavior provides evidence of a reversible, temperature-responsive reorganization of the ligand corona. At low temperature, it supports an anisotropic (lamellar-like) NP arrangement, whereas at elevated temperature the organic shell becomes effectively “molten”, favoring a more isotropic, short-range ordered structure. Overall, this first stage confirms that the mixed DDT/LC shell is both thermally robust and conformationally flexible—features that, as mentioned above and demonstrated previously,^63^ are essential once the NPs are embedded in the LC host.

As it is challenging to probe NP–BPLC interactions directly in the final BPLC composites (where NPs form only a small fraction of the volume), we next prepared thin-film AuNPs@DDT/LC–BPLC composites with 0, 5, and 50 wt % BPLC. These samples can be relatively easily tested by TEM, UV–Vis, and SAXS, reporting on whether the two components mix and on how the BPLC matrix modulates NP packing (Figure 4, Supporting Note 6, Table S7, Supporting Information).

**Figure 4:**
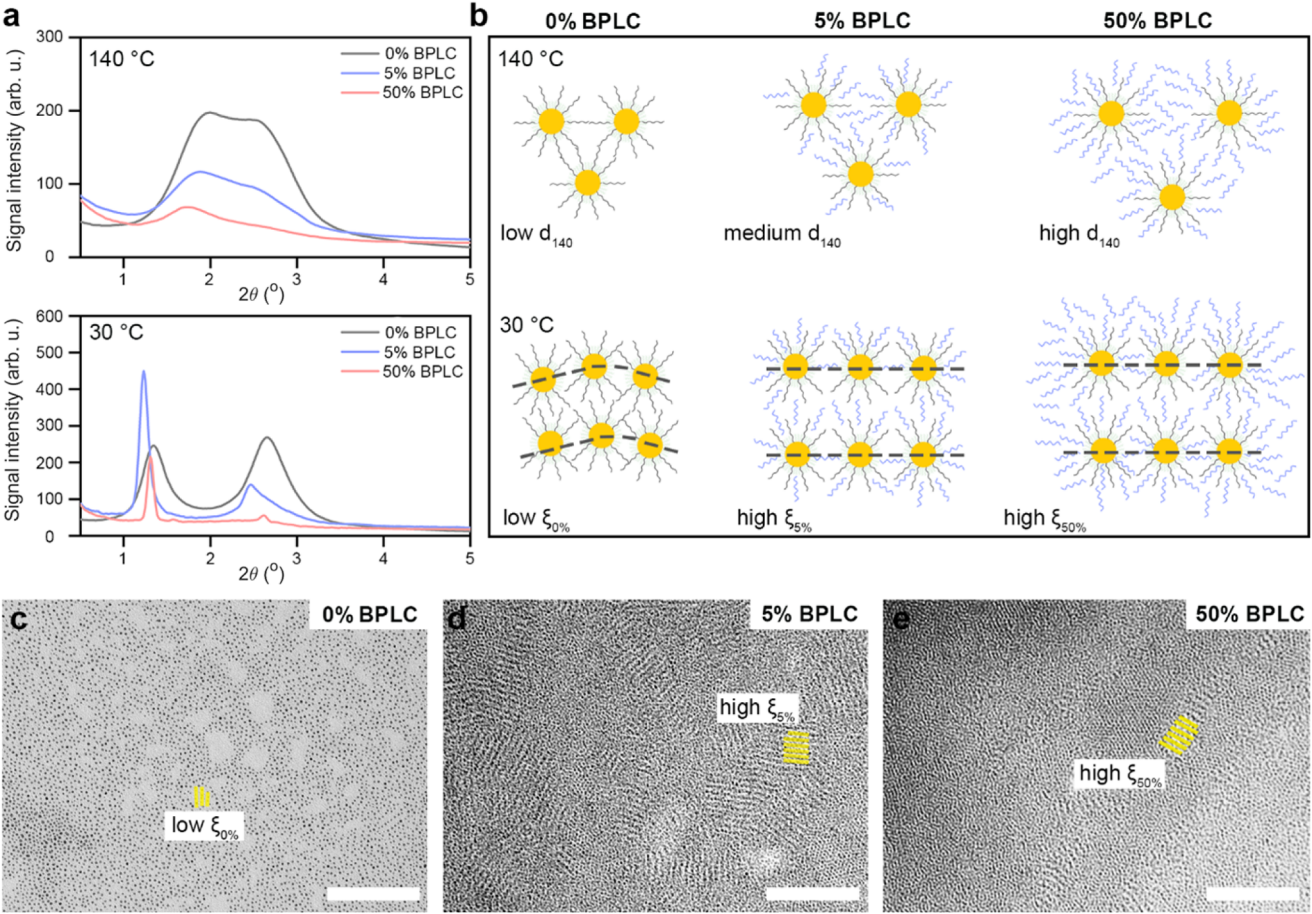
Analysis of internal effectors—NPs. (a) Small-angle X-ray scattering (SAXS) profiles of AuNPs@DDT/LC (0 wt % of BPLC) and AuNPs@DDT/LC-BPLC composites (5 and 50 wt % contents of BPLC). Measurements were collected at 140 and 30 °C, and the results are shown in the upper and lower diffractograms, respectively. Detailed positions of the peaks are given in Table S7 (Supporting Information). (b) Scheme of AuNPs@DDT/LC and AuNPs@DDT/LC-BPLC composites assembly, derived from the SAXS measurements. At elevated temperatures (upper row), the assembly is close to isotropic, with increased interparticle spacing as the amount of BPLC increases (the interparticle spacing at elevated temperature, 140 °C, is referred to as d_140_; the described tendency is reflected using low/medium/high d_140_). At lower temperature (lower row), the assembly is lamellar; the dashed curves in the lower row correspond to the interlayer distance in the lamellar-type assembly of the NPs; the curved line for the pure AuNPs@DDT/LC sample (i.e., the sample not doped with BPLC) indicates a shorter correlation length of the layered system (ξ), in contrast to systems doped with BPLC. (c–e) TEM micrographs of heat annealed AuNPs@DDT/LC and AuNPs@DDT/LC-BPLC composites, corresponding to the low temperature structures shown in panel b; the yellow lines serve as visual guides to indicate layer-type structures formed by NPs; the greater number of correlated layers is observed in samples doped with BPLC, qualitatively consistent with the increasing ξ in these samples. The white scale bar corresponds to 100 nm.

TEM at 30 °C reveals an intricate evolution of the NP arrangement (Figure 4c–e). Without BPLC, the particles form only short-range ordered fragments separated by more loosely packed regions. Adding 5 wt % BPLC results in larger, more uniform domains with locally lamellar/hexagonal ordering, indicating that the BPLC material penetrates the corona and helps homogenize interparticle spacing on the 10–100 nm scale. At 50 wt % BPLC, the assemblies become somewhat more heterogeneous again, with NP-rich domains separated by BPLC-rich regions; however, the particle size and contrast remain uniform, and no large clusters or sintered objects are observed. This nonmonotonic evolution, that is maximal coherence of particle arrangement at intermediate BPLC contents and slight relaxation at 50 wt%, strongly suggests that BPLC is chemically compatible with the LC-like ligands and modulates NP packing. While at high BPLC loading, the system starts to display partial mesoscale segregation of NP-rich and LC-rich regions rather than macroscopic phase separation.

SAXS further quantifies these trends (Figure 4a). At low temperatures, the correlation peaks sharpen as the BPLC concentration increases from 0 to 5 wt%, mirroring the improved domain coherence observed in TEM, and broaden slightly at 50 wt%, reflecting increased mesoscale heterogeneity. At 140 °C, when both the ligand shell and BPLC are molten, with increasing BPLC fraction, the main SAXS maxima systematically shift to lower angles, corresponding to larger average interparticle distances (d increasing from ∼4.4 nm to ∼5.1 nm). This trend indicates that in the molten state, BPLC efficiently infiltrates the corona and dilutes NP–NP contacts. Specifically, AuNPs@DDT/LC and BPLC are highly miscible at elevated temperatures, which is crucial for distributing particles throughout the BPLC volume prior to recrystallization.

Finally, UV–Vis spectra of thin films of AuNPs@DDT/LC show that introducing BPLC yields a narrower, blue-shifted LSPR at ∼504 nm, resembling the UV–Vis spectra of particle dispersion value than the NP-only films (Supporting Note 6). This indicates reduced plasmonic coupling and larger average particle separations when BPLC infiltrates the corona, in agreement with the above-discussed structural examinations.

In summary, TEM, SAXS, and UV–Vis collectively demonstrate that our internal effectors, AuNPs@DDT/LC, are

- thermally and colloidal durable,
- possess a compliant organic shell that mixes very well with BPLC at elevated temperatures, and
- that the organic shell can be partially infiltrated by BPLC molecules at low temperatures.

These properties are precisely those required for NPs intended to remain durable, disperse uniformly within the BPLC host at elevated temperatures, and preferentially localize within the defect network upon blue-phase formation. Notably, these properties of LC-coated NP-LC template interactions are in line with those reported in a recent study.^64^

### Fine-Tuning of the Photonic Properties of Quasi-Monodomain Soft Crystals

Based on the above-designed and chosen external and internal effectors, 12 samples were prepared in the following steps using four ALs (BY, PVA, PI-A, SE130) and three levels of NP doping on the BPLCs (0, 0.5, and 2 wt%)—this assessment aimed to evaluate the impact of the ALs and NPs. As a note, limiting the NP loading in LC materials below a few wt % is usual, as phase separation can be expected above such levels.

At the beginning of implementing our approach, we cooled the samples from the isotropic phase to the BPII phase. Figure 5a includes POM images of the monodomain textures of these samples, characterized by distinct selective light reflections at specific wavelengths. The observed reflection color depends on the type of ALs employed and the concentration of Au NPs. In other words, the surface anchoring energy (*external effector*) and the concentration of the NPs (*internal effector*) enable variation of the photonic bandgap. Below, we analyze the variation in detail.

**Figure 5:**
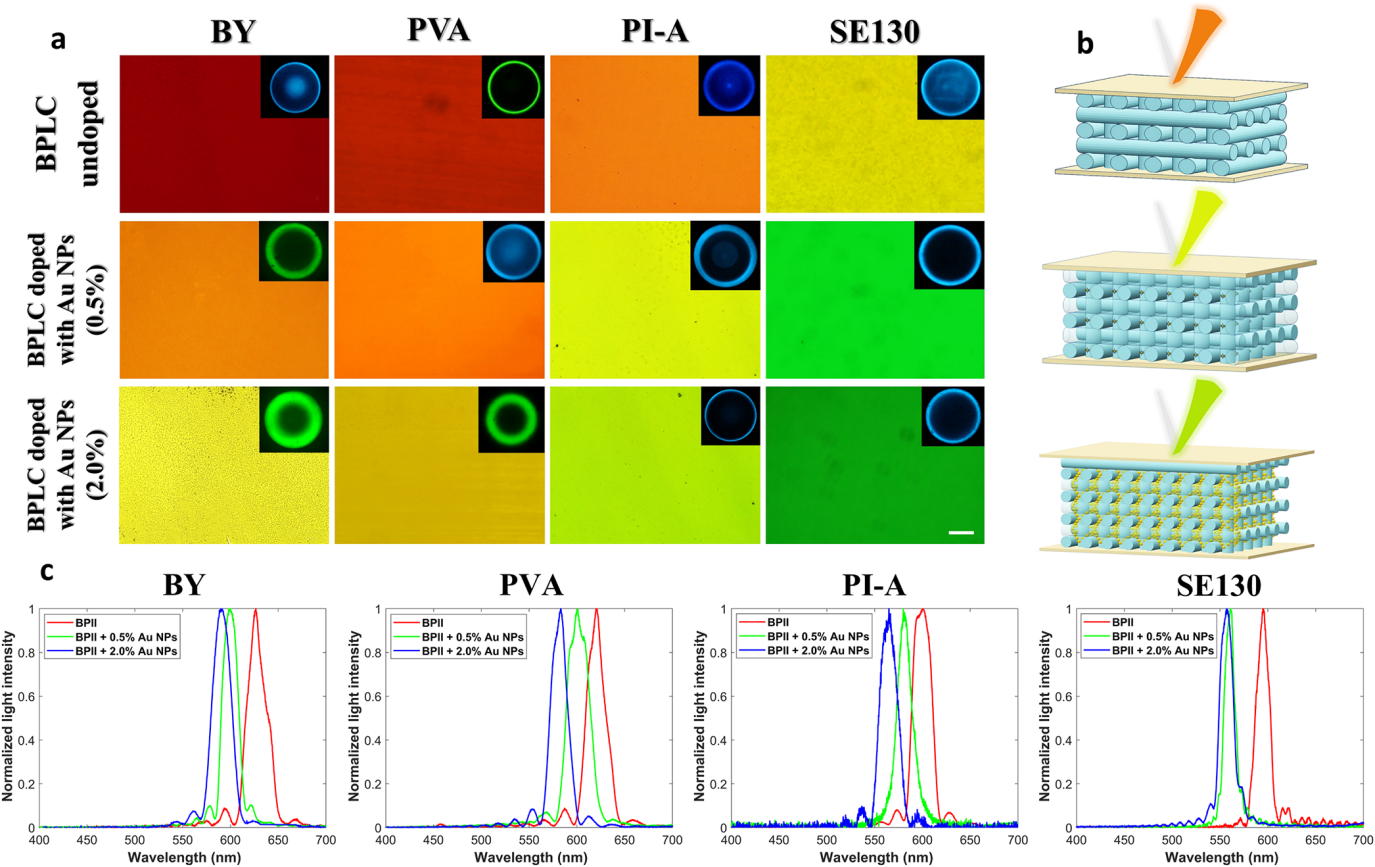
Fine-tuning of the photonic bandgap in BPII. (a) POM images of monodomain crystals in BPII. Selective reflection from the BPLC sample is controlled by applying homogeneous alignment layers with distinct anchoring energies and varying concentrations of gold nanoparticles. Insets show Kossel diagrams at 448 and 560 nm wavelengths, confirming the (100)-lattice orientation in BPII. The white scale bar corresponds to 200 μm. (b) Illustration of BPLC cells in BPII_(100)_ with PI-A alignment layers, depicting molecular self-organization of DTC structures at different gold nanoparticle concentrations. (c) The reflection spectra signals measured for NP-doped BPLC with each alignment layer in BPII exhibit a blue shift attributable to doping at higher concentrations.

In the case of undoped samples (Figure 5a, first row), the wavelength of the Bragg reflection transitions from a deep dark red light in the sample with BY AL through light red and orange hues for PVA and PI-A ALs up to a yellow reflection for SE130 AL. Kossel diagrams of these samples are shown as insets in the POM images, clearly depicting the characteristic donut-shaped pattern indicative of the (100)-lattice orientation of BPII, which is consistently maintained across all samples. These results imply two conclusions. First, the blue shift caused by the ALs is continuous with the growing surface anchoring energy of ALs. Second, we can speculate that the phenomenon is directly attributable to an increased chiral twist, i.e., a reduction in the helical pitch of DTC structures. Such a claim should be further strengthened by excluding potential artifacts that could cause the Bragg shift. On the one hand, a minor inclination of the BP lattice orientation relative to the (100) plane parallel to the substrate could produce such a variation, but then, the Kossel diagrams would exhibit an asymmetrical pattern,^65^ which is not the case here. On the other hand, potential minor deviations in the sample thickness were also shown to cause the variation. However, such deviations should be relatively large—smaller ones, on the order of ± 0.5 μm, which we expect for the LC cells used, do not significantly influence the Bragg reflection.^66^ Overall, we can conclude that the observed variation of the Bragg peak is best explained within the frames of the variable anchoring energy of the ALs.

In the case of 0.5 wt% Au NPs doped samples, incorporating NPs results in a pronounced shift in the Bragg reflection toward shorter wavelengths for each examined type of AL.

They preserve the continuous shift previously noted for the ALs, and these achiral functionalized Au NPs enhance the chiral twist within DTCs by introducing local elastic perturbations and molecular interactions. Specifically, LC-like ligands on the NP surface strongly bind via S–Au interactions and interact with the LC matrix through π–π stacking and dipolar interactions, which locally increase the helical twisting power (HTP) and favor configurations with shorter pitch. This effect is further amplified because NPs accumulate within the disclination network of the BP and interact with LC molecules at the disclination boundaries.^56^

When comparing samples doped with 0.5 and 2.0 wt % Au NPs, the acquired POM images attest that the extent of blue-shifting increases with the NP concentration (see Figure 5b). For the lowest azimuthal anchoring energy (i.e., BY ALs), the reflection color changes from red to orange to yellow upon NP doping. For the highest anchoring energy (i.e., SE130 ALs), the reflection color changes from yellow to green. These results are consistent with the idea that at higher NP concentrations, the LC-like ligands of the NPs infiltrate the DTC structures more effectively, thereby modifying elastic constants and reducing the equilibrium pitch. Consequently, the size of the BP unit cells decreases, resulting in a blue shift in the selective light reflection. Here, the growth of grain boundaries in the texture as defects can also be observed, indicating a mismatch in BP domain orientation over a large area of the sample (compare textures for PI-A or SE130 in Figure 6). However, this is not due to particle aggregation; the investigated NPs remain well dispersed up to 2.0 wt% in BPs. This is further confirmed by measurements of white-light transmittance spectra through the BPLC-doped samples in the isotropic phase (Figure S7, Supporting Information).

**Figure 6:**
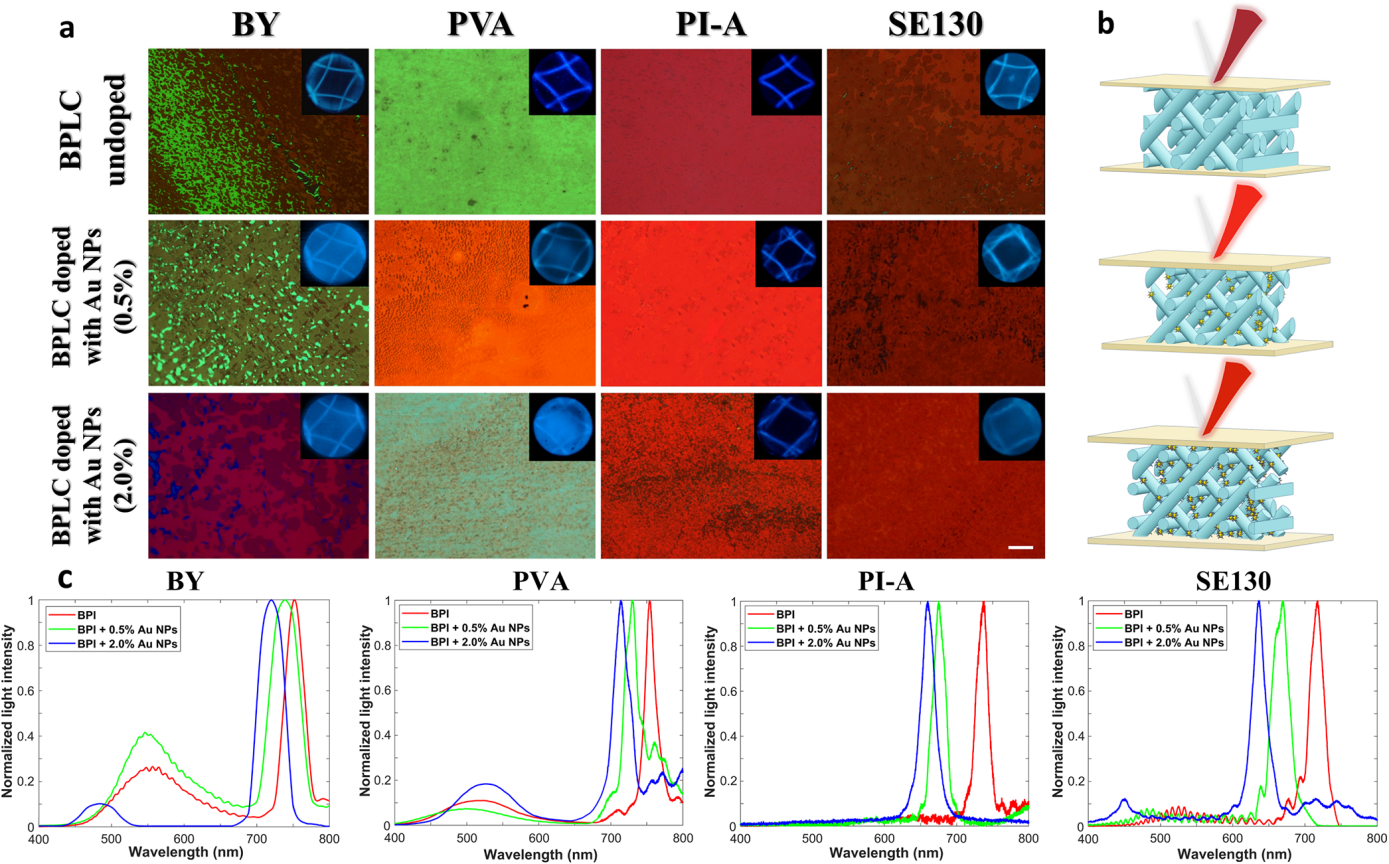
Fine-tuning of the photonic bandgap in BPI. (a) POM images of quasi-monodomain crystals in BPI. Selective reflection from the BPLC sample is controlled by applying homogeneous alignment layers with distinct anchoring energies and varying concentrations of gold nanoparticles. Insets show Kossel diagrams at 448 nm wavelength, confirming the (110)-lattice orientation in BPI. The white scale bar corresponds to 200 μm. (b) Illustration of BPLC cells in BPI_(110)_ with PI-A alignment layers, depicting molecular self-organization of DTC structures at different Au NP concentrations. (c) Reflection spectra signals were measured for BPLC samples with each alignment layer in BPI, indicating a blue shift due to NP doping.

The aspects described here are more qualitative in nature. A quantitative analysis based on the spectral measurements of the reflected light from BPLC samples in BPII (Figure 5c), along with the results for BPI, will be discussed later.

In BPI, most of our samples exhibit a polycrystalline nature. It is commonly noted in the literature that such organization of BP domains reflects the anchoring energy on the sample surface (see the first row of images in Figure 6a). As expected, when the anchoring energy is lower, the texture becomes more polycrystalline, displaying distinct light reflections in two colors. Conversely, with enhanced surface anchoring, the sample manifests a quasi-monodomain state, reflecting light in a single color. Only the dominant signal recorded in the reflection was considered for further analysis.

The Kossel diagrams depicted in the POM images in Figure 6a clearly show a 2-fold diffraction pattern, indicative of the (110)-lattice orientation of BPI. In contrast to BPII, the Kossel diagram measurements reveal a noticeable alteration in pattern size, although the color change in the POM images remains barely distinguishable due to its occurrence in the deep red spectrum. By comparing the Kossel patterns obtained at the same wavelength (i.e., 448 nm), it becomes evident that an increase in surface anchoring energy correlates with an enlargement of the Kossel pattern. A similar correlation is observed when comparing Kossel patterns for BPLC samples doped with varying concentrations of Au NP, using the specific AL. In each case, the patterns exhibit mirror symmetry, suggesting that the variation in diagram size is associated with changes in the BP lattice dimension. Thus, we can conclude that the incorporation of Au NPs induces a reduction in the helical pitch, which translates to a decrease in the unit cell size of BPI_(110)_, as exemplified by the BPLC samples with PI-A ALs (Figure 6b). This interpretation is further supported by spectral measurements of the reflected light from BPLC samples in the BPI phase (Figure 6c).

Furthermore, our observations are consistent with theoretical calculations performed for chiral nematics, as demonstrated by Kiselev and Sluckin.^67^ Their analysis shows that the anchoring conditions at the LC-cell surfaces play a fundamental role in determining the equilibrium twist configuration. They demonstrated that stronger anchoring leads to more significant changes in the helical structure, including the pitch wavenumber, which ceases to be a local minimum of the free energy surface once the initial configuration becomes unstable. As a result, transitions between metastable states with different half-turn numbers become increasingly relevant. This theoretical framework supports the experimental observation that increasing anchoring energy, whether through the chemical composition of the alignment layer or through nanoparticle doping, can modulate the helical pitch and thereby influence the lattice parameters of BPI.

A quantitative analysis of the BPI lattice constant variation based on spectral and Kossel diagram measurements, including a comparison between experimentally obtained and theoretically predicted Kossel diagrams, is detailed later in the text.

### The Benefits of the PI-A Alignment Layer

At this point, the unusual properties of the PI-A alignment layer introduced in this work are worth noting. This layer combines photoresponsive units with amide and imide functionalities. Thus, we expect the photoalignment process to facilitate a uniform interaction between the amide and imide groups of the AL and LC molecules. Indeed, PI-A ALs yielded a monodomain texture with minute defects in the BPI despite having lower anchoring energy than SE130 ALs (Table S4, Supporting Information), which did not produce such a uniform sample. However, the uniformity of the BPLC samples in BPI using PI-A aligns with theoretical calculations by Fukuda and Žumer,^62^ indicating that obtaining an ordered texture in BPI_(110)_ is possible for surface anchoring energies above 10^–5^ J·m^–2^. Moreover, as the photoalignment process affects the structure of the AL, it can also influence the magnitude of the surface anchoring interaction with LC molecules by adjusting the optical power of blue light during the exposure process in the preparation of the ALs on the glass substrates. This provides an additional factor for Bragg wavelength shifting in both the N* and BP phases, although the magnitude of the shift differs in each LC phase. An exemplary difference in Bragg reflection from BPLC samples with PI-A ALs, which differ only in the optical power density of blue light during the exposure process, is demonstrated in Figure S8, Supporting Information. Such Bragg wavelength control is not achievable with standard photoalignment layers, which lack amide/imide groups and have lower anchoring energies (<10^–5^ J·m^–2^).

An additional observation based on the recorded textures is that the type of alignment process (i.e., rubbing and photoalignment) is much less critical for influencing the Bragg wavelength shift. Instead, anchoring energy is the essential factor. Nevertheless, selecting an appropriate type of ALs may remain crucial for producing BP monocrystals over large volumes with a distinct Bragg reflection for specific BPLC materials.

### Quantitative Assessment of Photonic Properties Control

Beyond the above-discussed qualitative analysis, we wish to analyze the results in more detail and quantitatively. We obtained Bragg wavelengths from all 12 samples at various temperatures during cooling from the isotropic phase by measuring reflection spectra at a rate of 0.1 °C·min^–1^ (Figure S9, Supporting Information).

First, we focus on undoped samples and the impact of the *external effector*. As shown in Figure 7a, the Bragg wavelength shifts in the undoped BPLC material with the choice of AL type (surface anchoring energy). The reflection ranges from 595 to 627 nm (32 nm) for BPII and from 717 to 756 nm (39 nm) for BPI, with a blue shift as the surface anchoring energy increases. However, the induced shifts do not allow continuous tuning of the Bragg reflection across a wide spectral range, leaving a 90 nm spectral gap (627–717 nm; see the distance between the light red areas in Figure 7a).

**Figure 7:**
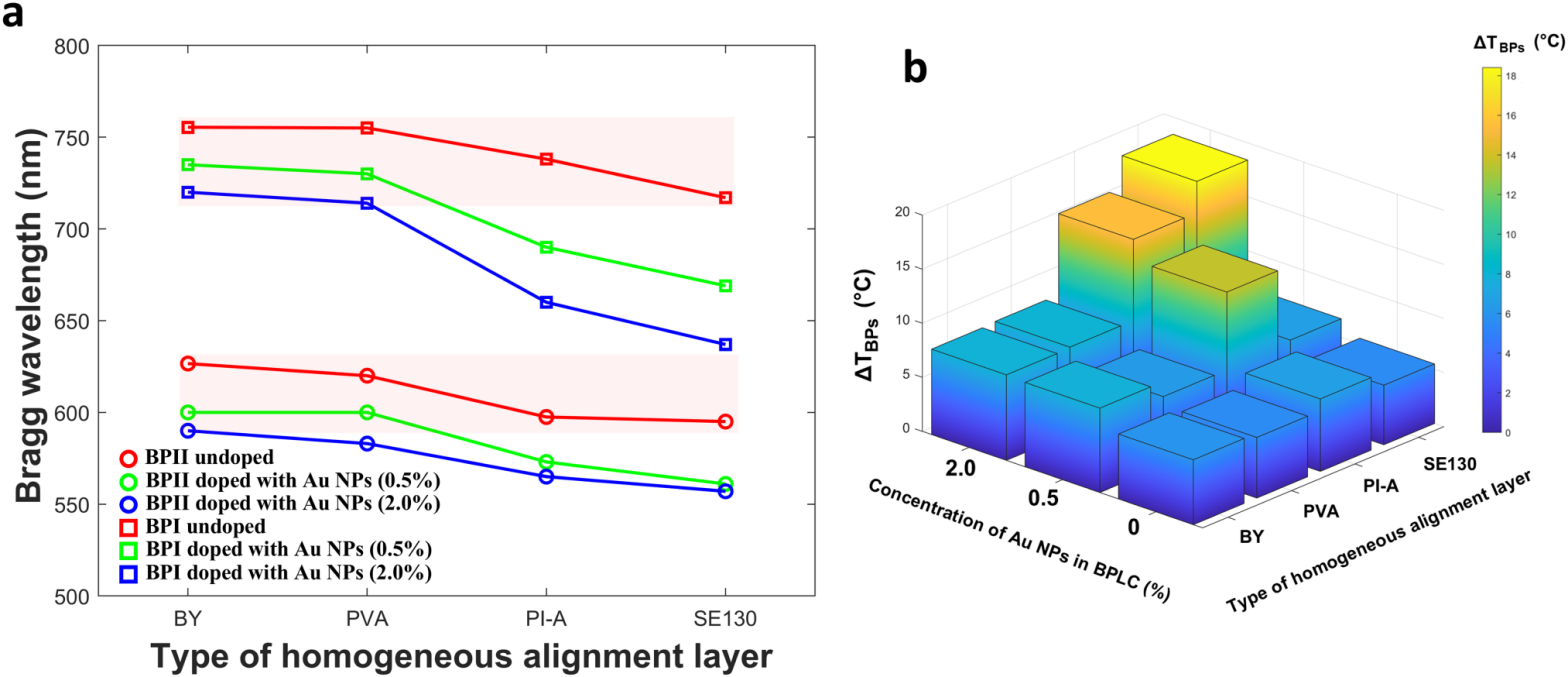
Overview of NP-doped BPLC #2 characteristics under varying surface anchoring energies. (a) Bragg wavelength value as a function of NP doping and the type of ALs used in the BPLC #2 sample. The light red rectangles indicate the limited range of photonic bandgap tuning achievable solely through different types of ALs, measuring 39 and 32 nm for the BPI and BPII regimes, respectively. (b) Thermal stability of BPs in BPLC #2, depending on the anchoring energy of ALs applied to the LC-cell substrates (type of ALs) and Au NP doping at various concentrations.

Second, let us analyze the effect of NP doping. For the weakest surface anchoring energy (BY case), the blue shift of reflection spectra in both BPs caused by NP doping is identical to that caused by ALs in undoped samples. Although this may be a coincidence, we hypothesize that the magnitude of the shift suggests strong (and similar) molecular interactions between SE130 ALs and BPLC to those between LC ligands and BPLC. Notably, the presence of amide groups and π–π interactions is likely to play a role in these interactions. Each of these molecules also possesses more pronounced interactions. The SE130 AL most probably supports stronger π–π interactions, which are balanced by the affinity of LC ligands toward the alkyl parts of the BPLC molecules, ensured by their aromatic-alkyl character.

Furthermore, we investigate whether the Bragg wavelength shifts induced by NP doping (up to 2 wt %) within the BPI and BPII structures are of comparable magnitude for the different AL types—that is, whether the effects of NPs and ALs are additive. Surprisingly, this is not the case. The magnitude of the NP-induced Bragg wavelength shift strongly depends on the AL employed. For BPII, the shift ranges from 37 to 38 nm, whereas for BPI it spans a much broader range, from 36 to 80 nm. The difference between ALs exhibiting the weakest (BY) and strongest (SE130) anchoring energy is therefore approximately 2-fold in BPI and remains small in BPII (compare the separation between the red and blue squares for BPI and the circles for BPII for the selected AL in Figure 7a).

Notably, the synergistic action of *external* and *internal effectors* ultimately allows for continuous control of the Bragg wavelength across both BPs within an overall range of approximately 200 nm (including 756 nm for the undoped BPI with the BY AL and 557 nm for NP-doped BPII with the SE130 AL; see Table S8 in the Supporting Information). Thus, the limitations of each individual approach are effectively overcome when combined and further supported by temperature control.

We also quantify the thermal stability distribution of the investigated BPLC samples, specifically the temperature range of BPs occurrence, depending on the used ALs (surface anchoring energy) and the level of NP doping. Figure 7b illustrates the thermal stability range for all tested samples, while detailed measurement data describing the thermal characteristics of the occurrence of each BP in the studied BPLC #2 samples are provided in Table S8 (Supporting Information). The results show that for relatively low anchoring energy (BY and PVA cases), the increase in thermal stability of BPs (i.e., BPI+BPII) due to NP doping is modest, from 6.0 to 7.9 °C for BY and from 5.6 to 8.0 °C for PVA. In contrast, for strong anchoring ALs (PI-A and SE130 cases), the enhancement in thermal stability of BPs due to NP doping is markedly greater, increasing from 6.7 to 15.5 °C for PI-A and from 5.5 to 18.4 °C for SE130. This represents an enhancement of approximately 2-fold for PI-A ALs and 3-fold for SE130 ALs, compared to the undoped BPLC samples. As in the case of Bragg reflection control, the thermal stability shift is synergistic, surpassing the additive calculations.

To fully appreciate the previously discussed Kossel diagram analyses, we compare the experimental results with the simulated Kossel diagram of a single unit cell in the (110)-lattice orientation of BPI. The Kossel lines were fitted to the experimental data at a wavelength of 488 nm (Figure 8a). Simulations revealed that, aside from minor variations in the azimuthal orientation of the crystal plane in BPI, the Kossel lines shifted further from the center of the diagram with increasing surface anchoring energy (from BY to SE130 ALs) and varying NP concentration, while still maintaining the (110) crystallographic orientation under both conditions. Uncertainty bars attached to the estimated lattice constant values in Figure 8b reflect the combined influence of these experimental factors, including the width and clarity of the Kossel lines and overall pattern quality (e.g., blurry or dim regions, nonuniform line thickness, and multiple intersecting lines). This uncertainty does not exceed ±12 nm.

**Figure 8:**
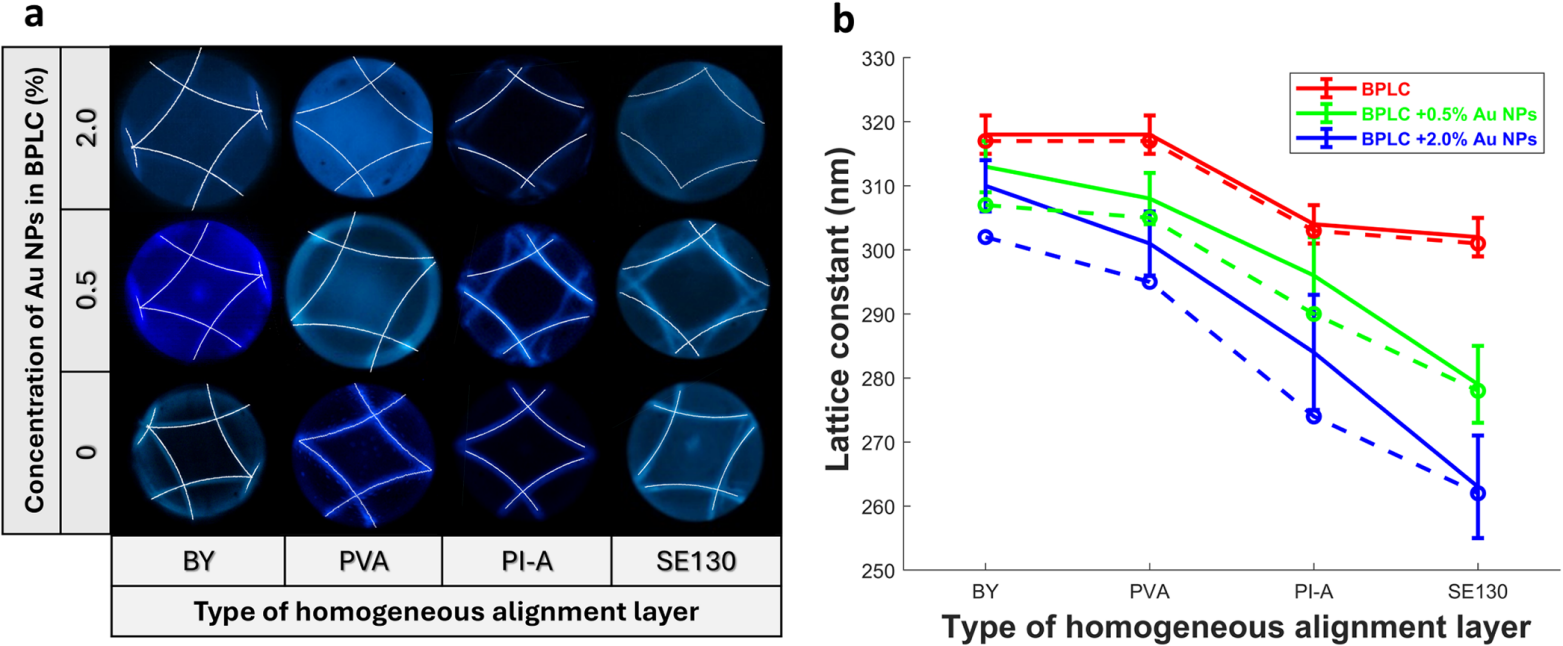
Analysis of the BPI lattice constant. (a) Comparison of the measured at 488 nm and simulated Kossel patterns (white lines) for BPI_(110)_ undoped and doped with Au NPs at various concentrations under ALs with different anchoring energies (i.e., the BPLC samples with varying types of AL). (b) Lattice constant of the (110)-oriented BPI structure as a function of AL, measured from Kossel diagrams (solid line) and calculated based on Bragg wavelength and average refractive index of BPLC measurements (dashed line).

These findings indicate a significant reduction in the cubic lattice constant due to strong interactions between LC molecules from DTC structures and anchoring ALs or ligands with LC-like properties on the surface of Au NPs. The lattice constant in BPI_(110)_ can be tuned using these factors within a range of approximately 60 nm (from about 260 to 320 nm). Simulated Kossel patterns are depicted in Figure S10 (Supporting Information). Additionally, our calculations of the lattice constant based on the Bragg wavelength equation for BPs showed good agreement with Kossel diagram measurements (Figure 8b), with a maximum difference of 12 nm. The theoretical two-approach analysis of the BP lattice constants considered a change in the mean refractive index of BPLC (*ñ*) due to NP doping, in line with our previous studies.^56^ The *ñ* values for undoped and NP-doped BPLC samples at concentrations of 0.5 and 2 wt % were 1.6773, 1.6941, and 1.7120, respectively, for BPI_(110)_, and 1.7024, 1.7047, and 1.7101 for BPII_(100)_. Detailed information and comparison of the lattice constant values for BPI_(110)_, including all examined BPLC composites, are presented in Table S9 (Supporting Information). Ultimately, the results presented here suggest an enhancement of the chiral twist in DTC structures, driven by varying surface anchoring energies resulting from ALs and NP doping in the BPLC material.

### Benchmarking Photonic Bandgap Engineering Techniques

Achieving precise control over the lattice size of molecular 3D self-assembly systems, such as in BPLCs, and thereby the photonic bandgap, is highly desirable for designing advanced photonic devices that operate within specific spectral ranges. However, this may be challenging. Here, we summarize the latest results of photonic bandgap engineering in BPLCs, as presented in Figure 9. To illustrate the effectiveness of various techniques in precisely controlling the Bragg wavelengths over the widest possible spectral range (Λ), we included a few essential statistical descriptors in the graph. Among them is the standard deviation (*σ*_Δλ̅_) that measures the dispersion of Bragg wavelength shifts around the mean, indicating the precision of control. A lower *σ*_Δλ̅_ suggests more consistent and reliable control over the wavelength shifts. While the mean (Δλ̅), median (Δλ̂), and mode (Δλ_o_) of Bragg wavelength shifts offer insights into the central tendency and distribution of the shifts, helping to identify the most typical and frequent changes. Together, these descriptors provide a detailed understanding of the performance and reliability of various techniques in controlling Bragg wavelengths, which is crucial for optimizing photonic devices. This detailed comparison highlights which techniques offer the most precise and reliable control over Bragg wavelengths. The method for determining the effectiveness of photonic bandgap control on several example data sets is presented in Figure S11 (Supporting Information).

**Figure 9:**
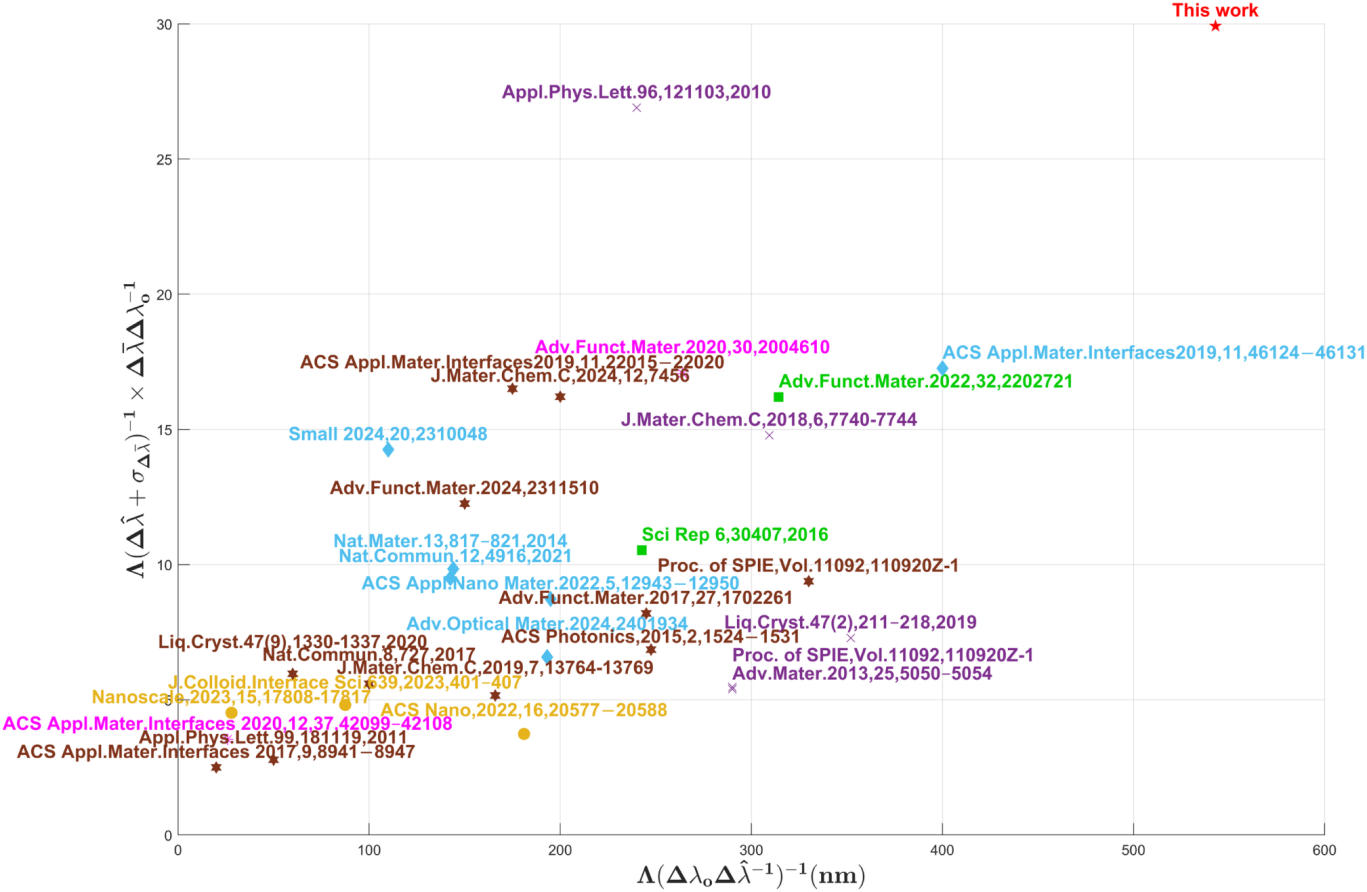
Overview of photonic bandgap engineering techniques. This figure compares various photonic bandgap tuning methods reported in the literature and our work. The techniques include nanomaterials doping (yellow circle), chemical functionalization or specific confinement geometry of the BPLC microstructure (green square), mechanical deformation of polymer-stabilized BP films (blue diamond), reaction with chemical substances (pink asterisk), application of electric fields (brown star), and optical fields (violet times). Our work (red star) involves using homogeneous alignment layers with different surface anchoring energies and gold nanoparticle doping at various concentrations. The factors on both axes are Λ represents the entire range of photonic bandgap tuning, while Δλ̅, Δλ̂, Δλ_o_ and *σ*_Δλ̅_ correspond to the mean, median, mode and standard deviation of the measured Bragg wavelength shifts, respectively.

Our approach, utilizing functional nanomaterials and ALs, ensures the achievement of long-term stable BPLC structures with controlled photonic bandgaps over a broad visible range of approximately 200 nm within a specific BPLC material. This process provides fine-tuning of the color reflection with a slight Bragg wavelength shift of approximately 5 nm. This is accomplished through NP doping at various concentrations and the application of ALs with distinct surface anchoring energies. At the same time, the cubic lattice orientation of the soft crystals within each BP is maintained, thereby preventing uncontrolled shifts in the Bragg wavelength as the lattice size changes under the influence of NPs and ALs. This method enables precise and stable tuning of photonic properties and may facilitate the development of applications with diverse requirements.

## Conclusions

In this work, we demonstrated the potential of BPLCs as attractive 3D chiral nanoarchitectures capable of exhibiting desirable photonic properties through the combined influence of alignment layers and nanomaterials. In other words, our study revealed two distinct categories of effectors that enable fine-tuning of the Bragg wavelength in BPLCs. The first effector (external) involves modulation of the chiral twist in BPLCs through interactions with chemical moieties in the ALs, which exhibit varying surface anchoring energies. The second effector (internal) leverages interactions between LC molecules from DTC structures and nanoparticle surface ligands with LC-like properties, which pack within the BPLC disclination network. These findings expand current understanding of how interfaces regulate chirality in thin-film organic materials.^68^ The role of effectors has been contrasted with conventional control methods, demonstrating their advantages. Moreover, the mechanistic role of each effector was studied in detail, providing both qualitative and (partially) quantitative insights into how the molecular design of ALs and NPs translates into their effective interactions with the BPLC matrix. This analysis outlines considerations for molecular design strategies to tailor photonic responses in BPLC systems.

Furthermore, we confirm that in both external and internal effector cases, stronger interactions lead to a reduction in unit-cell size, thereby altering the lattice constant while preserving the cubic orientation and symmetry of the soft crystallographic structures independently within each BP phase (BPI and BPII). Notably, this modulation occurs without reconfiguration of the defect network, as evidenced by the unchanged Kossel patterns, despite possible scaling effects.

Overall, the approach presented here enables precise photonic bandgap control (<10 nm) across a wide visible range (∼200 nm) in BPLCs. This capability not only enables deterministic adjustment of the reflected wavelength but also exceeds typical telecom requirements (≲10–20 nm in the C/L bands), aligning with the much broader spectral demands of advanced display technologies, even though challenges such as scalability and uniformity in NP–BPLC composites remain. These findings highlight molecular self-assembly of multicomponent nanomaterials as a powerful strategy for photonic bandgap engineering and for developing tunable BPLC-based devices operating within targeted wavelength ranges. The demonstrated tuning range is particularly relevant for color-selective displays and compact optical filters, where precise wavelength control is essential;^69^ it also supports wavelength-sensitive sensing for environmental and chemical detection. Incorporating nanoparticles can additionally enable responsive or switchable photonic elements for adaptive optics and smart-window technologies. Taken together, these advances establish a solid foundation for advanced photonic systems that integrate structural tunability with functional responsiveness.

## Experimental Section

### BPLC Material Synthesis

The BPLC materials were synthesized at the Institute of Chemistry of the Military University of Technology using components mainly developed in-house. The primary composition of the formulated BPLCs is a nematic mixture (85.8 wt %; additional details are provided in the Supporting Information) that comprises photochemically stable fluorinated oligophenyls with fluorinated cyclohexyl- and bicyclohexylbiphenyls.^70^ The compound structures and concentrations are listed in Table S1 (Supporting Information). The LC mixture was based on the low birefringence and temperature-dependent characteristics typical for nematics. The base mixture exhibits a dielectric anisotropy of Δε = 12.6 (measured at 1 kHz and 23 °C), an optical anisotropy of Δn = 0.178 (measured at 589 nm and 23 °C), and a rotational viscosity γ = 305 mPa·s. The BP phase in the investigated BPLC is induced by the inclusion of two chiral dopants (CDs) as follows: biphenyl-4,4-dicarboxylic acid bis­(1-methylheptyl) ester (CD #1) and [1,1;4,1] terphenyl-4,4-dicarboxylic acid bis­(1-methylheptyl) ester (CD #2).^71^ The measured macroscopic helical twisting power of biphenyl and terphenyl ester is 25 and 30 μm^–1^, respectively. To prepare three BPLC materials, which exhibit different Bragg reflections, the following CDs concentrations were added to the nematic mixture: 5.7 wt % (CD #1) and 5.4 wt % (CD #2) for BPLC #1, 7.0 wt % (CD #1) and 7.2 wt % (CD #2) for BPLC #2, and 8.7 wt % (CD #1) and 8.7 wt % (CD #2) for BPLC #3. Both structural formulas of the investigated CDs and their concentrations in the prepared BPLC mixtures are presented in Table S2 (Supporting Information). The helical pitch of the BPLCs under study, measured in the N* phase using the Grandjean-Cano method, was found to be 410, 350, and 280 nm for BPLC #1, BPLC #2, and BPLC #3, respectively, as demonstrated in Figure S1, Supporting Information.

### Nanoparticle Synthesis

The syntheses of nanoparticles and ligands were conducted following the previously described protocol.^72^

### Nanoparticle Characterization

The morphology and size distribution of the nanoparticles were examined by transmission electron microscopy (TEM) using a JEM-1011 microscope (JEOL, Tokyo, Japan) equipped with an EDS INCA analyzer (Oxford Instruments, Oxford, UK) at the Electron Microscopy Platform, Mossakowski Medical Research Center, Polish Academy of Sciences, Warsaw, Poland. TEM samples were prepared by drop-casting nanoparticle dispersions onto TEM grids, followed by solvent evaporation under ambient conditions. All other measurements were performed at the University of Warsaw.

Small-angle X-ray diffraction (SAXRD) measurements were carried out using a Bruker Nanostar system (Cu Kα radiation, parallel beam formed by cross-coupled Goebel mirrors and a 3-pinhole collimation system, VANTEC 2000 area detector; Bruker, Billerica, MA, USA). Samples for SAXRD measurements were prepared by drop-casting nanoparticle dispersions onto Kapton tape and allowing the solvent to evaporate.

UV–Vis spectroscopy measurements were performed using a GENESYS 50 UV–Vis spectrophotometer (Thermo Fisher Scientific, Waltham, MA, USA). Solution-state spectra were recorded for nanoparticle dispersions placed in optical cuvettes. Solid-state UV–Vis measurements were performed on samples prepared by drop-casting nanoparticle dispersions onto microscope glass slides, followed by solvent evaporation.

Thermogravimetric analysis (TGA) was performed using a NETZSCH STA 449 F3 thermal analyzer. Measurements were carried out under argon at a heating rate of 10 K·min^–1^ over the temperature range of 100–600 °C.

### Doping NPs into the LC Host

An NP dispersion in toluene was introduced into a glass vial containing a few milligrams of BPLC. Subsequently, the mixture was thoroughly mixed by sonication at room temperature for 2 min. The volume of the NP dispersion varied across different samples but was always more than 10 μL. The necessary volume of the NP dispersion was determined using an Au^0^ concentration, which was estimated from the sample’s absorption at 400 nm. After the mixing process, the vial was left under ambient conditions until the toluene had completely evaporated, which took at least 12 h.

### Azo Poly­(amide-imide) Material Synthesis

The azo poly­(amide imide) (PI-A) is a custom-synthesized material that was characterized in the previous work.^73^ The PI-A material showed a low molar mass, suggesting the material’s oligomeric nature (*M*_w_ = 6.2·10^3^ g·mol^–1^ (in DMF); *M*_w_/*M*_n_ = 1.9). Despite the low molar mass, the wide-angle X-ray diffraction patterns showed a single broad, diffuse diffraction peak in the 17–38° (2θ) range. The azo polyimide material exhibited a high glass transition temperature of 244 °C and excellent thermal stability, with a 5% weight loss temperature of 419 °C. UV–Vis spectroscopy revealed a broad absorption spectrum in the 300–500 nm range, with two λ_max_ at 317 and 345 nm in the film.

### Preparing BPLC Samples

In the experiment, 12-μm-thick cells were prepared on 0.7 mm-thick high-quality float glass plates. The glass plates were spin-coated with selected well-known materials, including a brilliant yellow azo dye (BY, Sigma-Aldrich), poly­(vinyl alcohol) (PVA, Sigma-Aldrich), polyimide SE130 (Nissan Chemical Industries, Ltd.), and a custom-synthesized azo poly­(amide imide) material (PI-A). The chemical structural formulas of all the used ALs are presented in Table S4 (Supporting Information). These materials were employed to achieve homogeneous alignment of LCs with various anchoring properties. The alignment materials were used either by rubbing (for SE130 and PVA) or by photoalignment (for BY and PI-A). Initially, the coated substrates were baked at 120 °C for 20 min for both BY and PVA, and at 80 °C for 30 min for SE-130. The latter required an additional 1.5 h of annealing at 180 °C. Subsequently, the substrates with PVA or SE130 were rubbed with low-pile velvet and assembled into glass cells in an antiparallel configuration using UV-cured micrometer balls as spacers. In contrast, the pair of substrates coated with BY or PI-A and assembled as glass cells were exposed to a linearly polarized beam: 457 nm at 10 mW·cm^–2^ and 445 nm at 150 mW·cm^–2^, respectively, from diode lasers at normal incidence. The irradiation time was 15 min for the BY-coated cells and 30 min for the PI-A ones. To investigate the influence of slightly varying anchoring energies on the photonic properties of BPLC, two samples with PI-A ALs were fabricated. This was achieved by using different optical power densities during irradiation: 80 vs 150 mW·cm^–2^. Photoinduced birefringence measurements were performed to confirm the successful photoalignment process after each exposure (Supporting Note 8). The empty glass cells were filled by capillarity with undoped and NP-doped BPLC materials at a high temperature corresponding to the isotropic phase for each sample.

### Contact Angle Measurements

Contact angle measurements for four ALs that induce homogeneous LC orientation were performed using a DSA100 goniometer (KRÜSS GmbH). Droplets of at least 3 μL of deionized water, the 1912 nematic LC, and the BPLC mixture were dispensed from a syringe needle onto the substrate surface. The substrate was then raised to make contact with the droplet, and the contact angle was measured on both sides of the droplet as the angle between the droplet’s tangent and the substrate. The final contact angle value was determined as the average of the angles measured on the left and right sides of each droplet. For nematic LC and BPLC, measurements were additionally performed as a function of temperature to account for phase-dependent wetting behavior.

### BPLC Sample Optical Characterization

BPLC textures were captured using a Nikon Eclipse LV100 POL polarizing optical microscope in reflection mode, equipped with a focusable, centerable Bertrand lens. An mK2000 temperature-controlled stage (Instec) facilitated the analysis of BPLC samples across a range of temperatures. Phase sequences of the LC samples, from isotropic to cholesteric phase, were studied at a cooling rate of 0.1 °C·min^–1^. It has been previously established that the cooling process yields blue phases over a more extensive temperature range than the heating process. Microscopic images and Kossel diagrams were captured using a DS-Fi1 charge-coupled device (Nikon) adapted for POM. Reflection spectra were obtained via a USB4000 spectrometer (Ocean Optics). For Kossel pattern analysis, BPLC samples were placed on a temperature-controlled stage and illuminated at 488, 560, or 650 nm with a 10 nm bandwidth. Kossel patterns were observed in the back focal plane of the objective (100×, NA = 0.9) in the microscope by inserting a Bertrand lens.

### Kossel Diagram Simulations

Utilizing Kossel-Kikuchi K-pattern simulation software, Kossel patterns were generated for single domains of the (110)-oriented body-centered cubic structure. The lattice constant of BPI was analyzed using monochromatic light at 488 nm. The simulated patterns were aligned with the experimental results, taking the refractive index of the BPLC samples into account. For comparison, lattice constants were indirectly estimated using the Bragg reflection equation for BPs.

## Supplementary Material



## Notes

Experimental details and additional data as noted in the text: helical-pitch analysis in the N* phase using a Grandjean–Cano wedge cell; photonic bandgap tuning versus chiral-dopant concentration with schematic reorientation of BPI/BPII cubic cells; temperature-dependent Bragg wavelengths for BPLC #2 with phase-resolved Kossel diagrams insets and representative textures; contact-angle images of nematic LC (1912) on BY, PVA, PI-A, and SE130 versus temperature; contact-angle images of BPLC #2 on BY and SE130 showing wetting changes across BPI/BPII; AuNP characterization before/after ligand exchange; verification of AuNP dispersion in BPLC cells by spatially repeated white-light transmittance spectra; Bragg-wavelength shifts versus photocuring power for PI-A alignment layers; temperature-dependent Bragg wavelengths for BPLC #2 for different ALs and NP doping levels; simulations of Kossel diagrams for BPI_(110)_ for undoped and NP-doped BPLCs across AL types; comparative framework for photonic-bandgap control under external factors; spatial stripes of monochromatic reflections created by a temperature gradient; wedge-cell confinement of BPLC domains; TGA traces of AuNPs@DDT and AuNPs@DDT/LC for ligand-coverage quantification; UV–Vis spectra of NP dispersions/films and NP–BPLC composite film; SAXS profiles of AuNPs@DDT and AuNPs@DDT/LC versus temperature with reversible superlattice rearrangement; photoinduced birefringence of PI-A; composition of the nematic mixture; chiral-dopant structures and concentrations for BPLC #1-#3; alignment-layer chemistries and azimuthal anchoring energies; thermal/photonics summary for BPLC #2 across ALs and NP loadings; comparison of BPI_(110)_ lattice constants from Bragg-equation estimates and Kossel-fit simulations (PDF)
