# Supplementary material for: Synergistic Effects of Nanoparticles and Surface Anchoring on Fine-Tuning the Photonic Bandgap in Blue Phase Liquid Crystals
Source: ACS Nano. 2026 Jun 8;20(24):17286–302. doi: 10.1021/acsnano.6c01127 (PMC13296597; doi:10.1021/acsnano.6c01127)
Supplement: Supplementary file 1 [file nn6c01127_si_001.pdf]

Supporting Information

Synergistic Effects of Nanoparticles and Surface Anchoring on Fine-Tuning Photonic Bandgap in Blue Phase Liquid Crystals

Kamil Orzechowski[1]*, Martyna Wasiluk[2], Weronika Milewska[1], Natalia Kowalska[2], Yi-Te Chuang[3], Jia-Yu Cao[3], Chun-Ta Wang[3], Aleksandra Neumann[1], Olga Strzeżysz[4], Anna Kozanecka-Szmigiel[1], Jolanta Konieczkowska[5], Ewa Schab-Balcerzak[5,6], Wiktor Lewandowski[2]*, Tomasz R. Woliński[1]

[1]Faculty of Physics, Warsaw University of Technology, Koszykowa 75 Str., 00-662 Warsaw, Poland

[2]Faculty of Chemistry, University of Warsaw, Pasteura 1 Str., 02-093 Warsaw, Poland

[3]Department of Photonics, National Sun Yat-sen University, No. 70 Lien-hai Rd., Kaohsiung 80424, Taiwan

[4]Institute of Chemistry, Military University of Technology, Kaliskiego 2 Str., 00-908 Warsaw, Poland

[5]Centre of Polymer and Carbon Materials, Polish Academy of Sciences, M. Curie-Sklodowska 34 Str., 41-819 Zabrze, Poland

[6]Institute of Chemistry, University of Silesia, Szkolna 9 Str., 40-007 Katowice, Poland

*Corresponding authors: kamil.orzechowski@pw.edu.pl, wlewandowski@chem.uw.edu.pl

Table of Contents

Table of Contents....................1

Supporting Figures....................2

Supporting Note 1. Structural organization of BPI vs BPII. 11

Supporting Note 2. Thermal gradient tuning of photonic bandgap in BPLC. 11

Supporting Note 3. Effect of anchoring gradient on BPLC domains within wedge geometry. 12

Supporting Note 4. Temperature dependence of anchoring energy and wetting behavior. 13

Supporting Note 5. Ligand quantification on nanoparticle surface. 14

Supporting Note 6. Absorbance measurements of nanoparticles. 15

Supporting Note 7. X-ray measurements of nanoparticles. 16

Supporting Note 8. Photoinduced birefringence measurements of the PI-A material. 17

Supporting Tables....................18

Supporting Figures

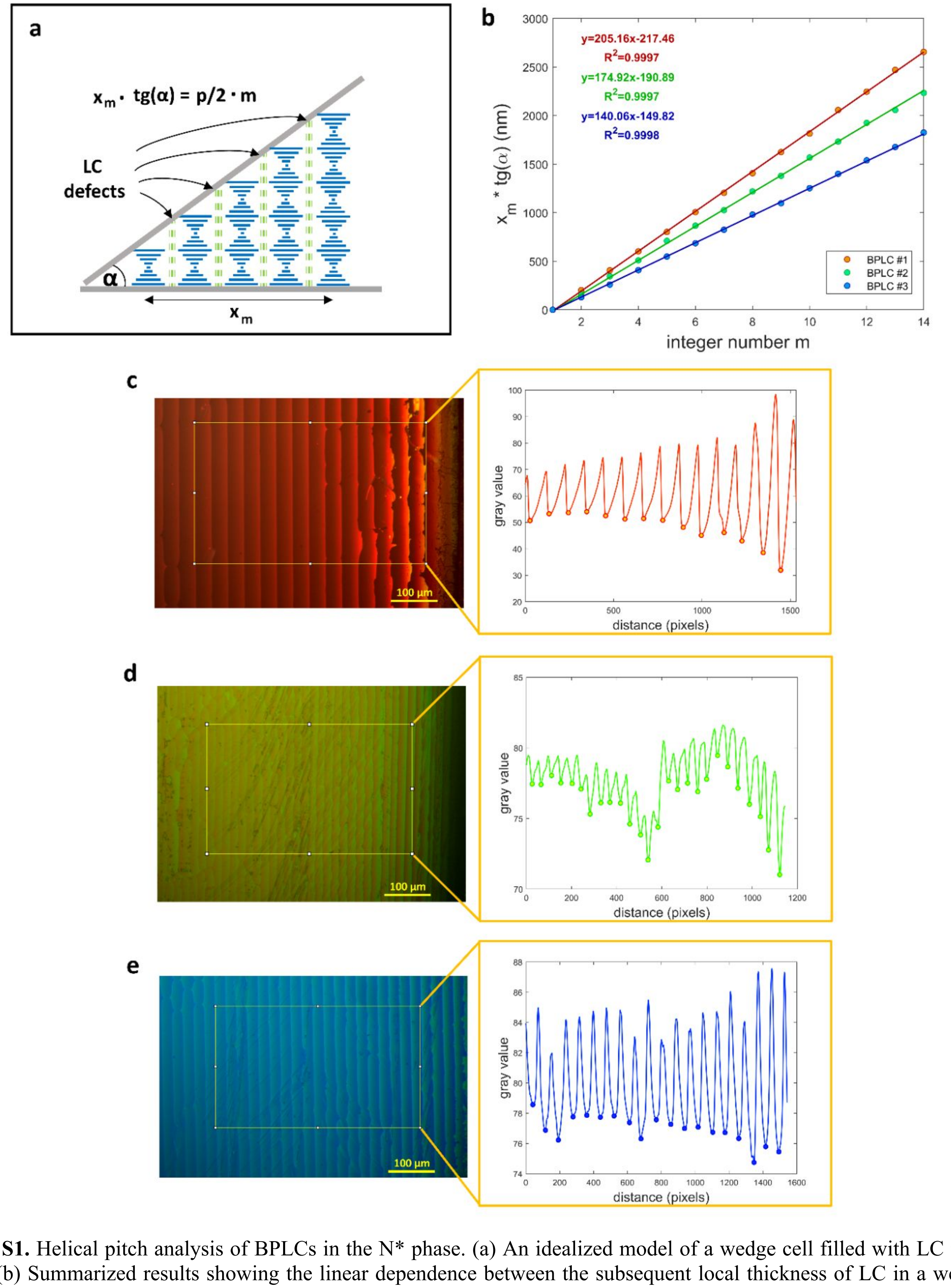

Figure S1. Helical pitch analysis of BPLCs in the N* phase. (a) An idealized model of a wedge cell filled with LC in the N* phase. (b) Summarized results showing the linear dependence between the subsequent local thickness of LC in a wedge cell, corresponding to an integer number *m* of half-pitches located in the gap, for three analyzed BPLCs. (c-e) The Grandjean-Cano wedge cell images of BPLCs in N* phase, with the graph of changing the gray value intensity of pixels along the width of the yellow rectangle from the images obtained for BPLC #1, BPLC #2 and BPLC #3, respectively. The rectangular selection in pixel intensity analysis provides a "column average plot", where the x-axis represents the horizontal distance through the selection

and the y-axis corresponds to the vertically averaged pixel intensity. The slope of the linear dependence indicates the half pitch of the investigated BPLC samples in the N* phase. The angle of each empty wedge cell was measured optically by considering the position of two reflected red laser spots from both plates of the wedge sample, and equals 0.30°, 0.57°, and 0.25° for BPLC #1, BPLC #2 and BPLC #3, respectively.

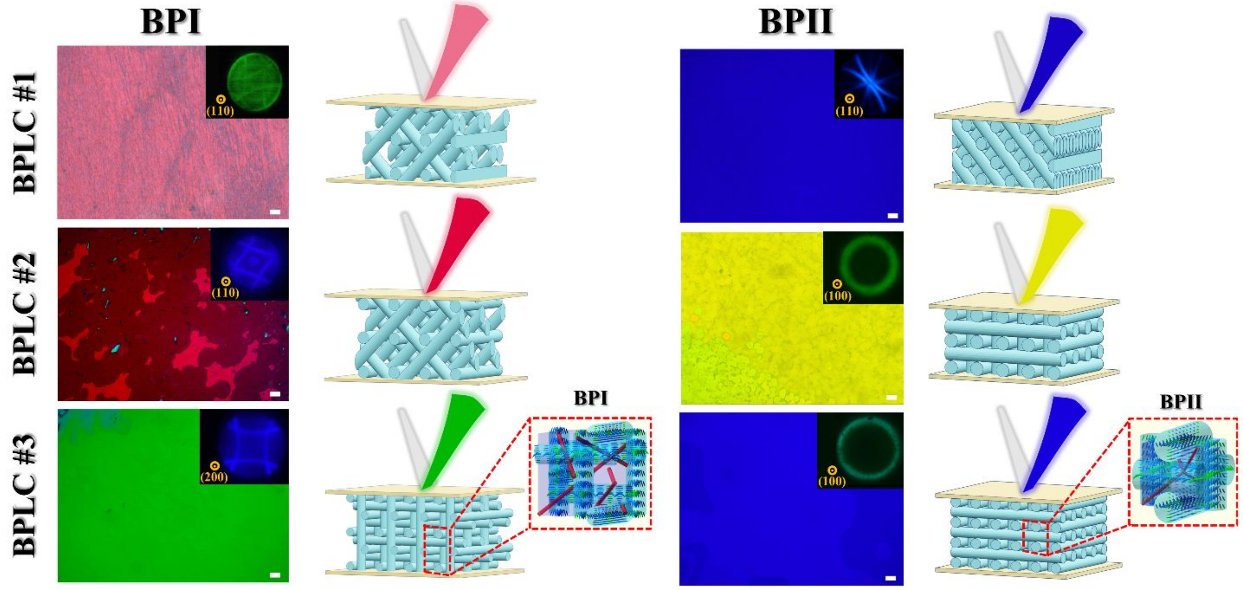

Figure S2. Photonic bandgap tuning in BPs using chiral dopants. For higher chiral dopant concentrations, the lattice size in BPs decreases. Additionally, the cubic unit cell reorients at distinctly different concentrations, as schematically demonstrated for BPI and BPII phases. The white scale bars correspond to 100 µm.

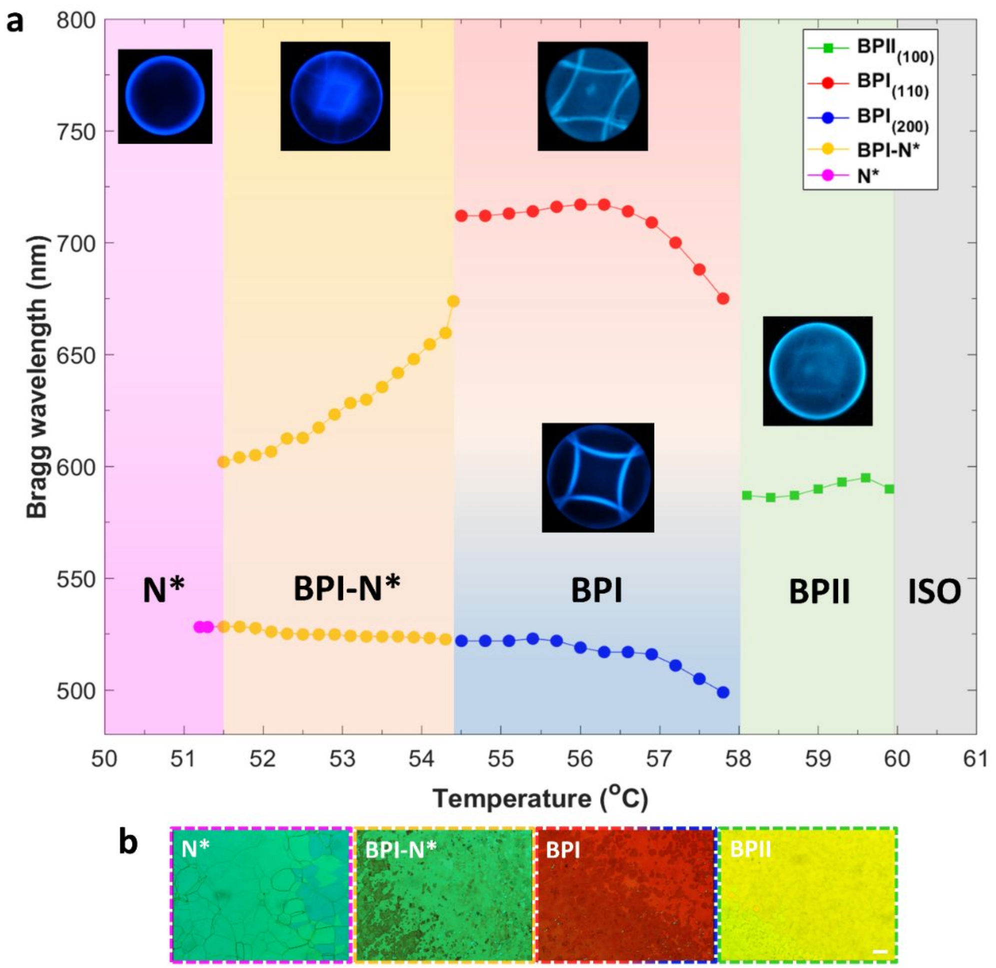

Figure S3. Temperature-dependent Bragg wavelengths for BPLC #2. (a) The graph illustrates the temperature dependence of the Bragg wavelength, measured during the slow cooling of the sample from the isotropic phase at a rate of 0.1°C·min^{-1}. Insets display Kossel diagrams measured at 448 nm, corresponding to different liquid-crystal phases. (b) Liquid-crystal textures were recorded at various temperatures, corresponding to each LC phase. The white scale bar represents 200 μm.

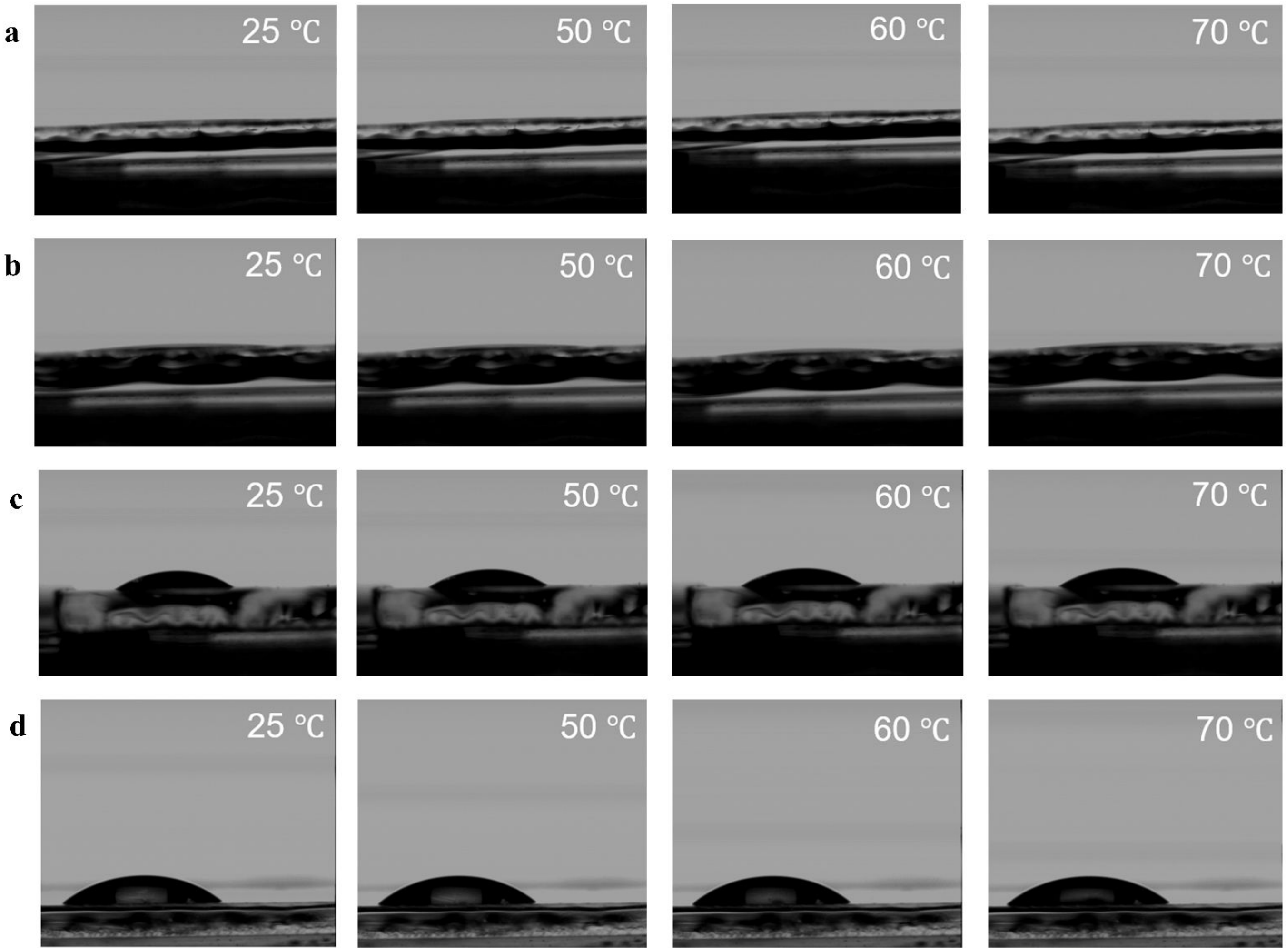

Figure S4. Images of the 1912 nematic LC droplet for contact angle measurements at different temperatures on the tested ALs: (a) BY, (b) PVA, (c) PI-A, and (d) SE130.

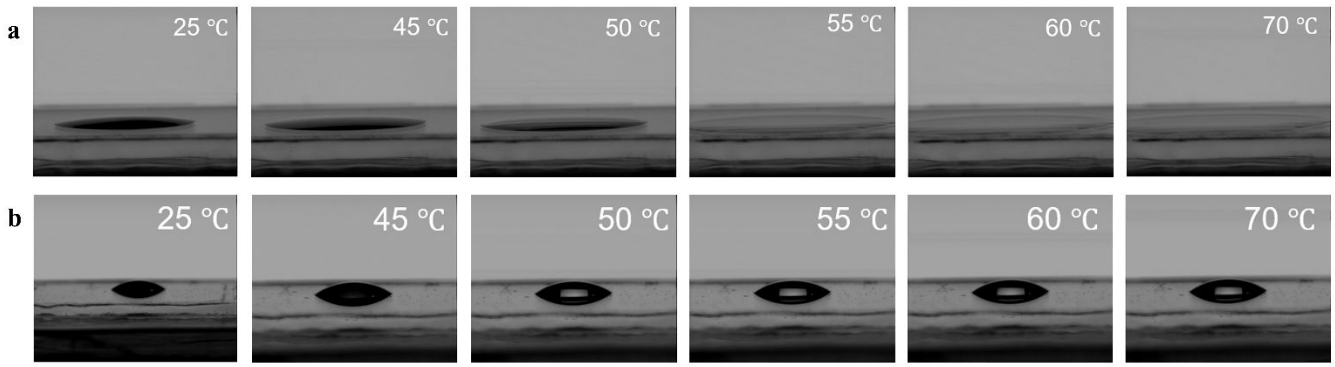

Figure S5. Images of the BPLC #2 droplet for contact angle measurements at different temperatures on ALs representing extremes of azimuthal anchoring energy: (a) BY and (b) SE130. These images illustrate the influence of phase transitions (including BPI and BPII) on surface interactions.

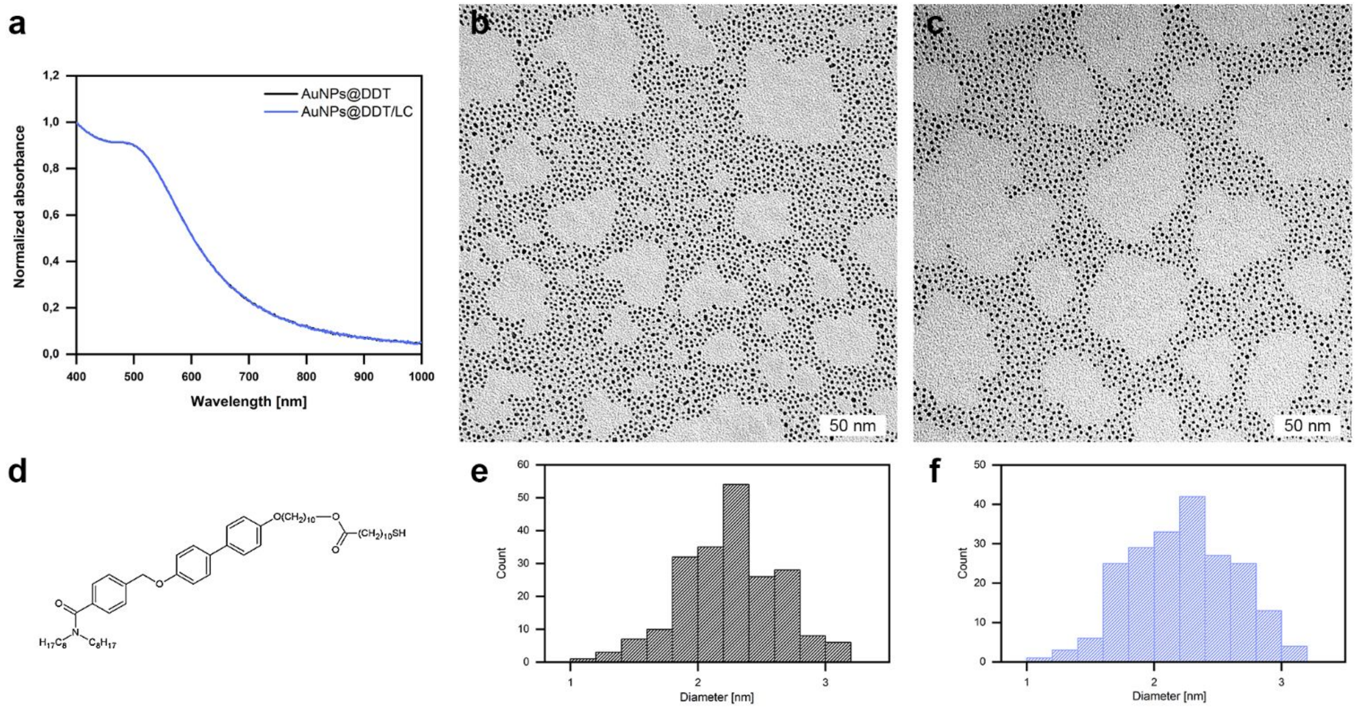

Figure S6. Characterization of the investigated NPs. (a) UV-Vis spectra of the NPs before (AuNPs@DDT, black) and after (AuNPs@DDT/LC, blue) ligand exchange. (b, c) TEM images of AuNPs@DDT and AuNPs@DDT/LC, respectively. (d) Chemical structure of the LC-like ligand ([(4′-{[4-(dioctylcarbamoyl)phenyl]methoxy}-[1,1′-biphenyl]-4-yl)oxy]undecyl 11-sulfanylundecanoate). (e, f) Size-distribution histograms for AuNPs@DDT and AuNPs@DDT/LC, respectively. The average diameter was (2.3 ± 0.4) nm for AuNPs@DDT and (2.2 ± 0.4) nm for AuNPs@DDT/LC.

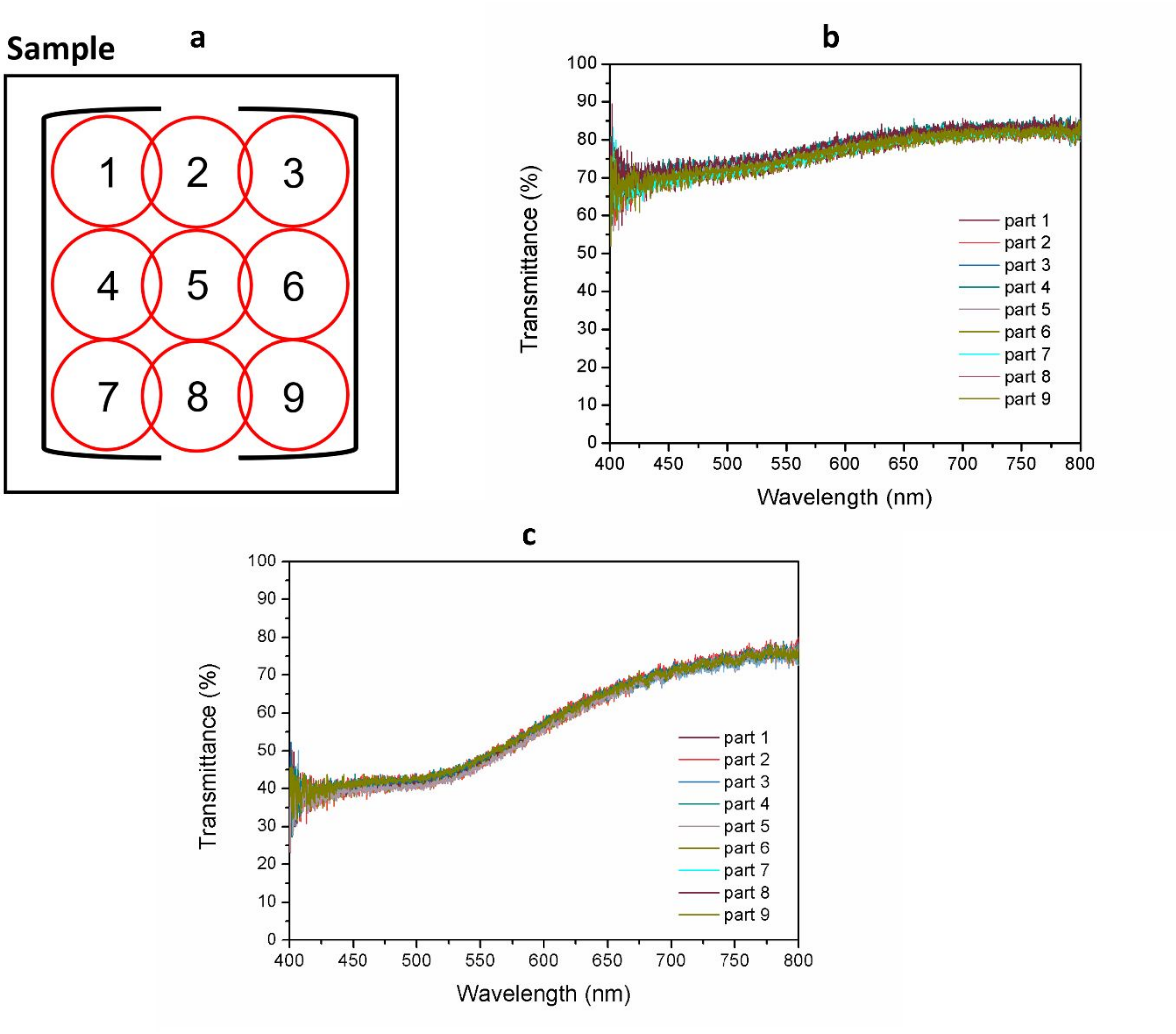

Figure S7. Study of NP dispersion in BPLC composites. (a) Illustration of the BPLC cell used. White-light transmittance spectra through NP-doped BPLC #2 samples with different NP concentrations: (b) 0.5 wt% and (c) 2 wt%. Measurements were performed in the isotropic phase and repeated at different locations within the LC cell to verify the uniformity of NP dispersion in BPLC. The graphs are presented for the BPLC #2 samples with SE130 AL, while they are identical for each of the studied ALs.

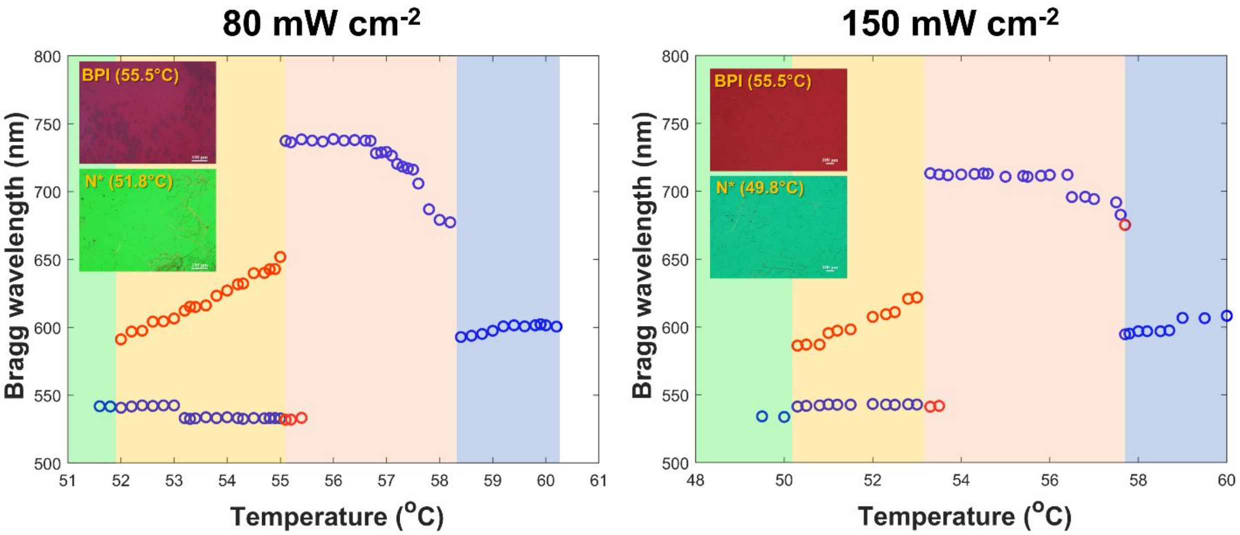

Figure S8. Bragg wavelength shift due to changes in photocuring conditions. Temperature-dependent Bragg wavelengths for BPLC cells with PI-A alignment layer exposed to blue light at optical power densities of 80 mW·cm^{-2} (left) and 150 mW·cm^{-2} (right). Higher optical power density during the photocuring of alignment layers results in greater surface anchoring energy, causing a blue shift in the Bragg wavelength, enhanced thermal stability, and lower phase transition temperatures. This shift is most pronounced in the BPI phase and least in the N* and BPII phases. In both cases, the irradiation time was 30 minutes. Blue and red open circles indicate the highest intensity of the reflection spectrum measured in a given LC phase for higher and lower optical signals, respectively. The green, light red, and blue regions in the diagrams correspond to the N*, BPI, and BPII phases, respectively, while orange indicates a continuous phase transition from the N* phase to BPI, where two distinct reflection peaks are achieved. Insets show the POM texture in BPI and N* phases, indicating a difference in the color of reflection due to the use of various optical densities of blue light during the exposure process. The investigations were carried out by cooling the samples from the isotropic phase at a rate of 0.1 ^{o}C·min^{-1}.

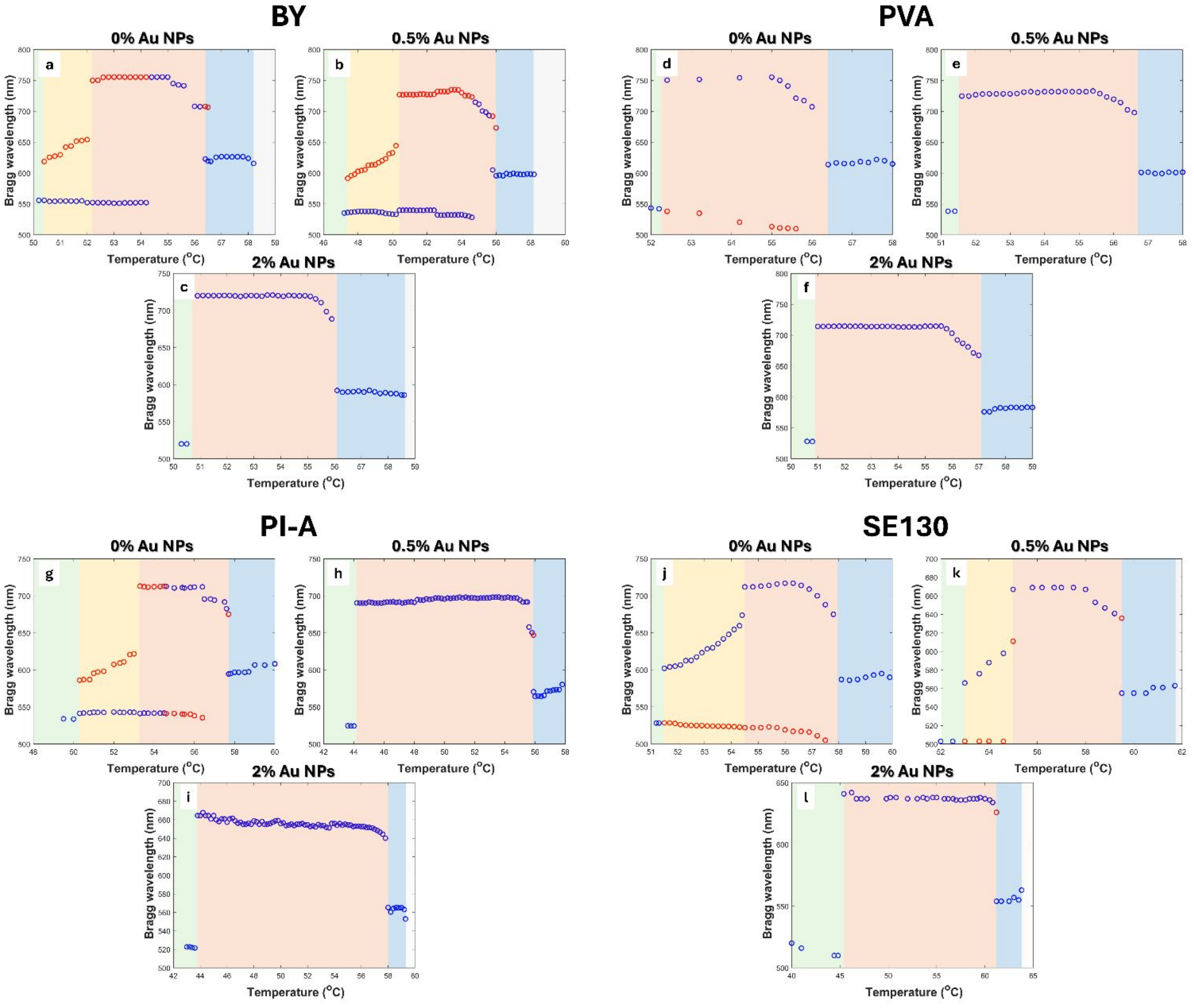

Figure S9. Temperature dependence of the Bragg wavelength during controlled cooling (0.1 °C·min^{-1}) in BPLCs with four homogeneous ALs (BY, PVA, PI-A, SE130) and three NP doping levels (0, 0.5, and 2 wt%). Panel layout: (a-c) BY with 0/0.5/2 wt% Au NPs, (d-f) PVA with 0/0.5/2 wt% Au NPs, (g-i) PI-A with 0/0.5/2 wt% Au NPs, (j-l) SE130 with 0/0.5/2 wt% Au NPs. Background shading indicates LC phases (green: N*, red: BPI, blue: BPII). The orange band marks the continuous N*-BPI transition regime, where two distinct reflection peaks coexist. Open circles mark the spectral maxima of the reflection band (blue: higher-intensity peak; red: lower-intensity peak).

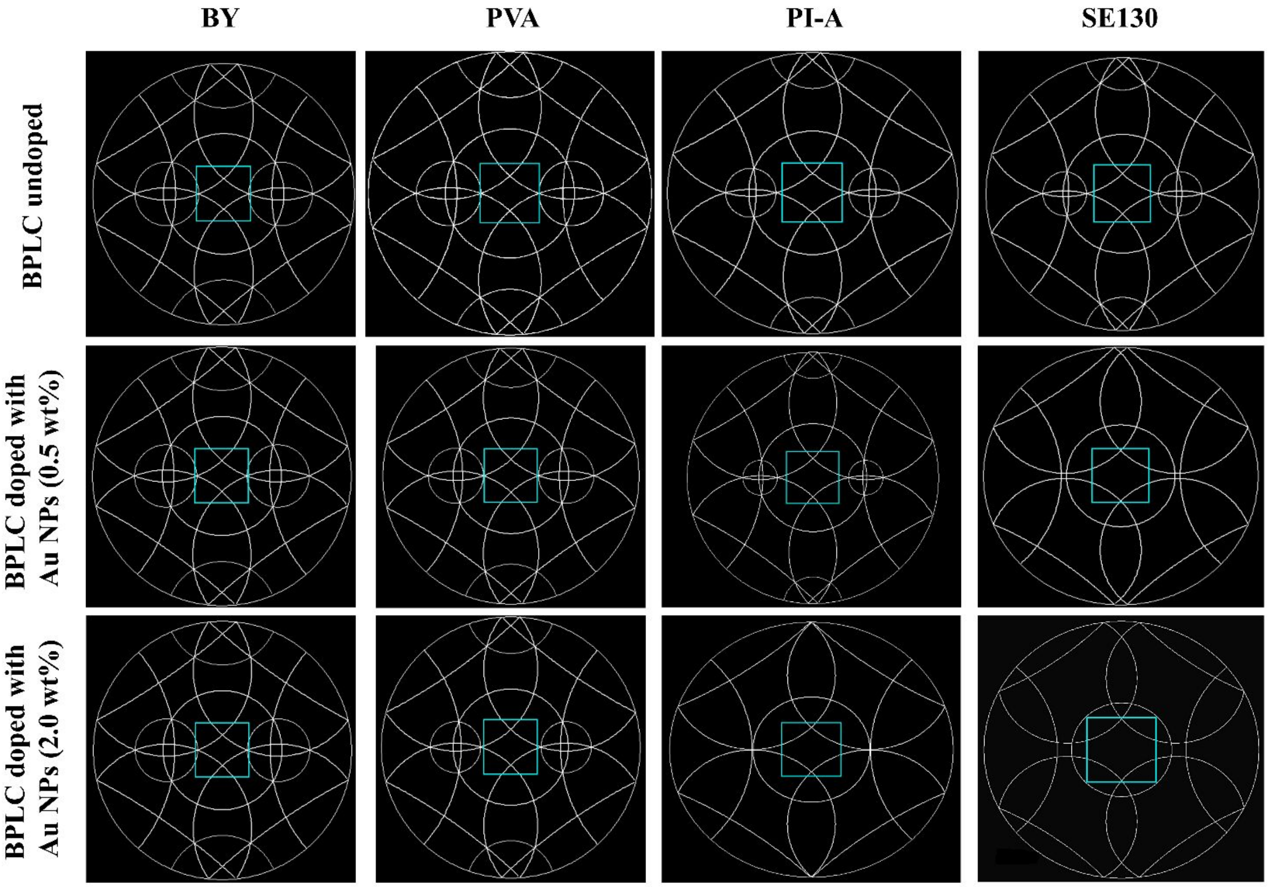

Figure S10. Simulations of the Kossel diagrams for $BPI_{(110)}$. The simulations were conducted for both undoped and NP-doped BPLC samples, with various ALs applied. The cyan square indicates the area observed under the microscope during the experiment. Simulations were performed at a wavelength of 488 nm.

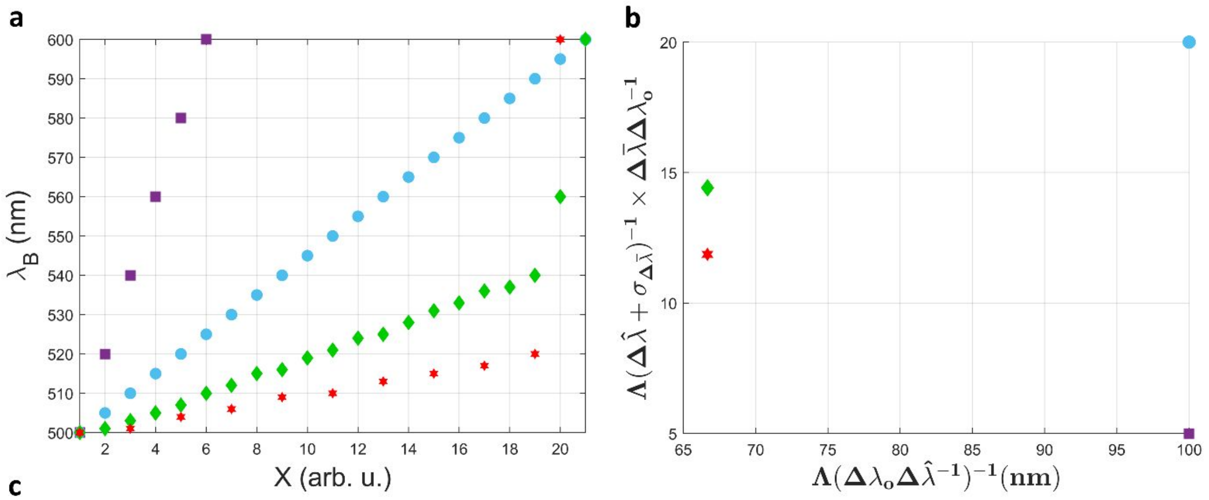

c

range, $\boldsymbol{\Lambda}$ (nm)	100	100	100	100
mean, $\boldsymbol{\Delta\bar{\lambda}}$ (nm)	20	5	4.76	9.09
std_dev, $\boldsymbol{\sigma_{\Delta\bar{\lambda}}}$ (nm)	0	0	9.01	23.54
median, $\boldsymbol{\Delta\hat{\lambda}}$ (nm)	20	5	2	2
mode, $\boldsymbol{\Delta\lambda_0}$ (nm)	20	5	3	3
$\boldsymbol{\Lambda(\Delta\lambda_0\Delta\hat{\lambda}^{-1})^{-1}}$ (nm)	100	100	66.67	66.67
$\boldsymbol{\Lambda(\Delta\hat{\lambda}+\sigma_{\Delta\bar{\lambda}})^{-1}\times\Delta\bar{\lambda}\Delta\lambda_0{}^{-1}}$	5	20	14.42	11.87

Figure S11. Potential photonic bandgap control in BPLC samples under various external conditions. (a) Examples of four data sets of Bragg wavelength shifts (Δλ) obtained by applying a specific external factor *X* (e.g., electric or optical fields, temperature, nanoparticle doping, or mechanical stress). (b) Effectiveness of Bragg wavelength shifts according to our approach. This includes precision, total spectral range of shifting, and other essential quantities. (c) Detailed numbers show the effectiveness of Bragg wavelength shifting achieved for the four chosen data sets.

Supporting Note 1. Structural organization of BPI vs BPII.

In general, the difference in the texture between BPI and BPII phases is attributed to the unique organization of the LC disclination network within the cubic unit cell. In BPII, all disclination lines converge at the center of the unit cell, prompting the BP crystallites to align and merge with adjacent ones, thereby forming a continuous defect network. This results in a single-crystal structure that represents a global free-energy minimum. In contrast, within BPI, the presence of multiple non-intersecting disclination lines per unit cell enables the relaxation of BP crystallites into their respective local energy minima, thereby promoting the maintenance of a polycrystalline state.

Supporting Note 2. Thermal gradient tuning of photonic bandgap in BPLC.

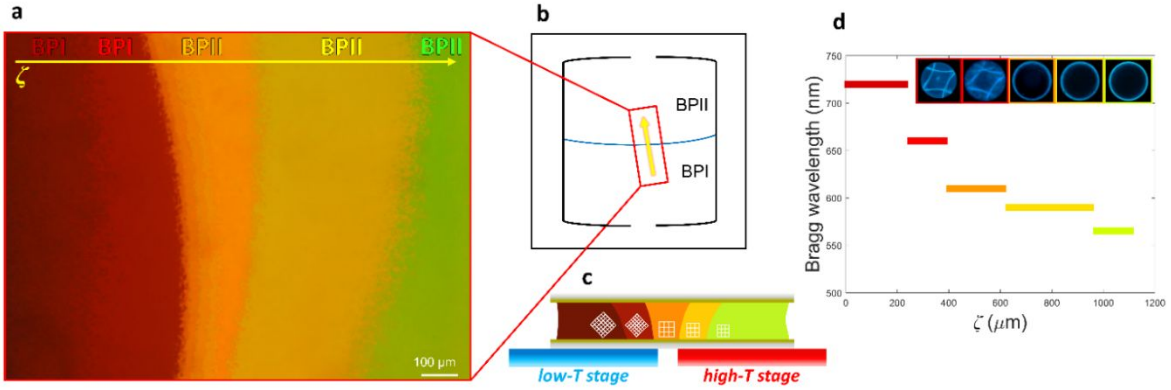

Figure S12. Multi-color reflection stripes created by a temperature gradient. (a) POM image showing colorful stripes resulting from reflections of monochromatic light, ranging from red to green, induced by a temperature gradient. (b) Schematic of the examined BPLC cell, with a yellow arrow indicating the direction along which the POM image was captured. The blue line marks the boundary between two LC phases (BPI/BPII). (c) Illustration of the side view of the BPLC cell placed on two heating stages at slightly different temperatures. The applied thermal gradient causes a spatial distribution of BP single crystals with multi-monochromatic light reflections, due to the temperature-dependent size of the crystal lattice (white cubic unit cells) in BPI (red regions) and BPII (orange, yellow, and green regions). (d) Graph of Bragg wavelengths measured along the yellow arrow in the POM image (horizontal position in the POM image, ζ). Insets show Kossel diagrams corresponding to each region probed at 448 nm.

In the BPLC #2 sample, an area of approximately 1.4 mm^2 exhibited a spatial distribution of monochromatic light reflections, ranging from red to green in narrow stripes (Figure S12a). This effect was observed after annealing the sample for several minutes at the phase transition threshold from BPII to BPI (Figure S12b), following slow cooling (0.1 $^{o}C \cdot min^{-1}$) from the isotropic phase. Metastable states in BPs were controlled by a temperature gradient within the BPLC sample, created by positioning it on two heating stages with slightly different temperatures, separated by 2 mm (Figure S12c).

The spatial variation in monochromatic light reflections is discontinuous, even though it corresponds to successive visible spectrum frequencies. The POM image reveals boundaries between adjacent homogeneous areas, highlighting the discrete nature of metastable states in BPs. Oriented BP crystals within each monodomain area exhibit thermal stability within a very narrow temperature range (a few tenths of a degree). Each domain is characterized by a specific lattice constant (or helical pitch). This behavior results from multiple local minima in the total free energy of BPLC, caused by the balance between twist energy (i.e., the equilibrium helical pitch) and surface anchoring energy, both temperature-dependent. However, the pitch and, consequently, the twist energy typically change rapidly with temperature.

Interestingly, the spatial modulation of monochromatic light reflections in these metastable BPs depends on the group symmetry of the cubic structures. Figure S12d illustrates a distinct discontinuous jump in Bragg wavelengths from orange (610 nm) to red (660 nm). This discontinuity arises for two reasons: (i) the coexistence of two BP phases (i.e., $BPII_{(100)}$ and $BPI_{(110)}$) due to temperature differences, and (ii) different minimum total free energy states within each phase. Reflections at 720 and 660 nm occur in the BPI region, while 610, 590 and 565 nm appear in the BPII region. Measured Kossel diagrams probed at each colorful stripe (added as insets in Figure S12d) unambiguously identify the corresponding symmetries and confirm the presence and spatial distribution of BPI and BPII. These observations provide insight into the structure and photonic properties of BPLC. The variation in twist energy must overcome the surface anchoring barrier for pitch jumps to occur, enabling the formation of metastable states with well-defined optical characteristics. Considering that the lattice constant of the unit cell in BPI is twice that of BPII, the difference between successive helical pitches in BPI is approximately twice that in BPII. Detailed calculations are presented in Table S3. A similar phenomenon has been theoretically analyzed and experimentally demonstrated for a single cholesteric LC material in the planar configuration[1–4].

Supporting Note 3. Effect of anchoring gradient on BPLC domains within wedge geometry.

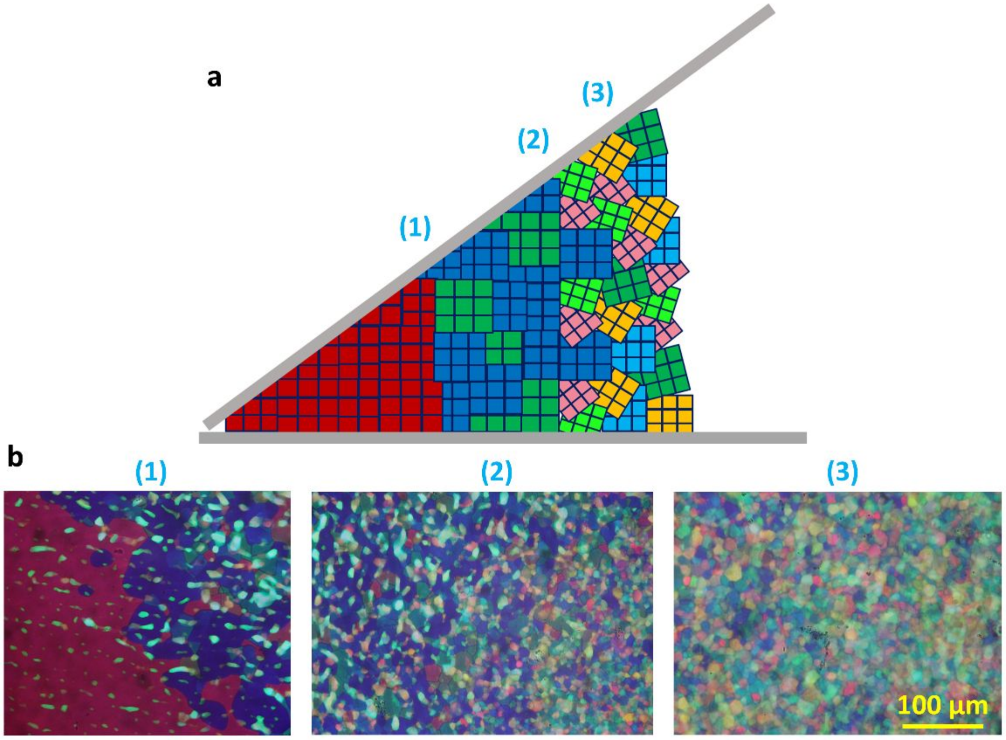

Figure S13. BPLC domains orientation under wedge confinement. (a) An illustration of a wedge cell with SE130 ALs filled with BPI domains. (b) POM images of the BPLC #2 were recorded at different thicknesses of the wedge cell. The results were obtained in transmission with the BPLC wedge sample between crossed polarizers to show the BP crystals forming within domains clearly.

Our results show that relatively large BP quasi-monodomains with a reflection color for a specified Bragg wavelength in a wedge cell can be achieved, albeit only at thinner sample thicknesses (area no. 1) up to around 20 μm and within a small area (~0.03 mm^2). It is essential to note that a monodomain texture was achieved here by using homogeneous alignment layers on both substrates (i.e., SE130). Conversely, at greater thicknesses (area no. 2), a Bragg wavelength shift occurs. Finally, when the surface anchoring energy is negligible (area no. 3; for approximately 200 μm-thick cell), the size of BP crystals significantly decreases (~0.0002 mm^2), becoming more polycrystalline and resulting in vivid and colorful reflections.

This phenomenon arises from the competition between homogeneous and heterogeneous nucleation during the BP crystal growth process. Homogeneous nucleation occurs in the bulk region of materials, leading to random crystal orientation, whereas heterogeneous nucleation takes place at the interface of LCs and glass substrates, resulting in crystals with ordered orientation. The proportion of BP crystals formed through heterogeneous nucleation is higher in the thinner regions of the BP wedge cell, thus leading to highly ordered crystal orientation. As sample thickness increases, the proportion of BP crystals formed through homogeneous nucleation rises, resulting in less ordered crystal orientations in the thicker regions of the wedge cell.

Supporting Note 4. Temperature dependence of anchoring energy and wetting behavior.

Temperature affects anchoring energy by reducing the order parameter of LCs, which generally leads to a weakening of molecular alignment and a decrease in anchoring strength. For BY and PVA, the nematic LC continued to spread completely across the surface at elevated temperatures, confirming persistent strong wetting and suggesting high polar anchoring energy.

Due to this, we were unable to determine the contact angle hysteresis proposed by Chibowski et al.[10] by measuring either the advancing or receding contact angles, which correspond to the movement of the liquid front of the settled drop as it advances or recedes, respectively. Consequently, it was not possible to obtain precise values of surface anchoring energies for those analyzed ALs.

However, slight decreases in the measured contact angles at elevated temperatures for PI-A and SE130 ALs, presented in Table S6, suggest improved wetting and modest increases in polar anchoring energy. Since the contact angle is inversely related to the work of adhesion, which reflects the strength of polar interactions, lower angles imply stronger polar anchoring. As temperature rises, however, the overall anchoring energy may still decrease due to thermal disorder, even if wetting improves slightly.

In systems with weak polar anchoring, the azimuthal component becomes more dominant, as noted by Muravsky and Murauski[11], where strong azimuthal anchoring ($W_\varphi > 10^{-5}$ J·m^{-2}) sets the lower limit of total anchoring, while weak azimuthal anchoring ($W_\varphi \leq 10^{-7}$–10^{-6} J·m^{-2}) makes polar anchoring the upper limit. The BY and PVA layers likely exhibit low azimuthal anchoring energy, allowing polar anchoring to dominate and resulting in complete wetting. In contrast, PI-A and SE130 may possess higher azimuthal anchoring energy, contributing more significantly to the overall anchoring, especially as temperature rises and the polar component becomes less influential due to reduced molecular order. Typically, polar anchoring energy is more sensitive to temperature changes, as it depends on the tilt angle of LC molecules

relative to the surface, which is directly affected by the decrease in the order parameter at elevated temperatures. In contrast, azimuthal anchoring tends to be more stable, since it is governed by the in-plane orientation of the molecules rather than their tilt, and is therefore less directly influenced by thermal fluctuations.

Moreover, our findings indicate that the presence of chiral dopants in the BPLC mixture does not significantly affect wetting behavior or polar anchoring energy, with variations of about ±1-2° compared to the nematic LC.

Supporting Note 5. Ligand quantification on nanoparticle surface.

To quantitatively assess the extent of ligand exchange, we performed thermogravimetric analysis (TGA) of the AuNPs@DDT (before ligand exchange) and AuNPs@DDT/LC (after ligand exchange). Measurements were carried out under argon at a heating rate of 10 $K \cdot min^{-1}$ in the 100–600 °C range. For AuNPs@DDT, a single mass-loss step was observed between 200 and 250 °C, which we assign to degradation of the dodecanethiol shell; the corresponding mass loss was 25.8%. In contrast, AuNPs@DDT/LC exhibit two distinct mass-loss steps: the first, also between 200 and 250 °C, amounts to 15.5% and is attributed to residual DDT, whereas the second, between ~300 and 450 °C, amounts to 15.1% and is assigned to degradation of the LC-like ligand. The nearly equal mass losses associated with DDT and the LC-like ligand indicate an approximately 1:1 mass ratio of the two ligands on the nanoparticle surface. Considering their molecular weights (202.4 $g \cdot mol^{-1}$ for DDT and 900.4 $g \cdot mol^{-1}$ for the LC-like ligand), this corresponds to a molar fraction of LC ligands of about 18%, i.e., a DDT:LC molar ratio of roughly 4.5:1. In other words, the mixed monolayer after ligand exchange contains approximately one LC-like ligand for every four to five DDT molecules. It is important to note that, before TGA, the samples were thoroughly purified after ligand exchange, so that detached DDT molecules and excess LC-like ligand that did not bind to the Au surface were removed; therefore, the TGA-derived masses reflect only ligands that remain bound to the nanoparticles.

To convert these mass losses into an approximate number of ligands per nanoparticle and per unit surface area, we assume spherical Au cores with an average diameter of 2.3 nm. The volume of a single core is then $V = 4/3 \cdot \pi \cdot (1.15 \times 10^{-7}\ cm)^3 \sim 6.4 \times 10^{-21}\ cm^3$, which, using the bulk density of gold ($\rho = 19.32\ g \cdot cm^{-3}$), gives a core mass of $m_{Au} \sim 1.2 \times 10^{-19}$ g and thus $\sim 8.1 \times 10^{18}$ nanoparticles per gram of Au. For the AuNPs@DDT sample, the TGA mass loss of 25.8% corresponds to 0.3477 g of DDT per 1 g of Au (0.3477 g / 202.4 $g \cdot mol^{-1} \sim 1.72 \times 10^{-3}$ mol of DDT for a 1 g of Au NPs), which, when including the number of NPs in gram, translates to **~127 DDT molecules per nanoparticle.** This accounts for a surface coverage of approximately **7.7 DDT per nm²** (using $S = 4\pi r^2 \approx 16.6\ nm^2$).

In the exchanged sample (AuNPs@DDT/LC), normalizing the organic mass losses to the residual Au mass gives 0.223 g DDT and 0.218 g LC-like ligand per 1 g of Au, corresponding to $n_{DDT} \sim 1.10 \times 10^{-3}$ mol and $n_{LC} \sim 2.42 \times 10^{-4}$ mol. This translates to **~82 DDT and ~18 LC-like ligands per nanoparticle**, i.e., ~100 ligands per 2.3 nm particle in total. With the same core surface area, this accounts for an overall coverage of **~6 ligands per nm²**, consisting of ~4.9 DDT and ~1.1 LC-like ligands per nm^2. Thus, ligand exchange

reduces the total coverage from ~7.7 to ~6 ligands per nm² and replaces about 36% of the original DDT molecules with bulkier LC-like ligands. This moderate decrease in overall coverage is fully consistent with the significantly larger molecular size and higher molar mass of the LC-like ligand and remains within the typical range reported for densely packed monolayers on small Au nanoparticles.

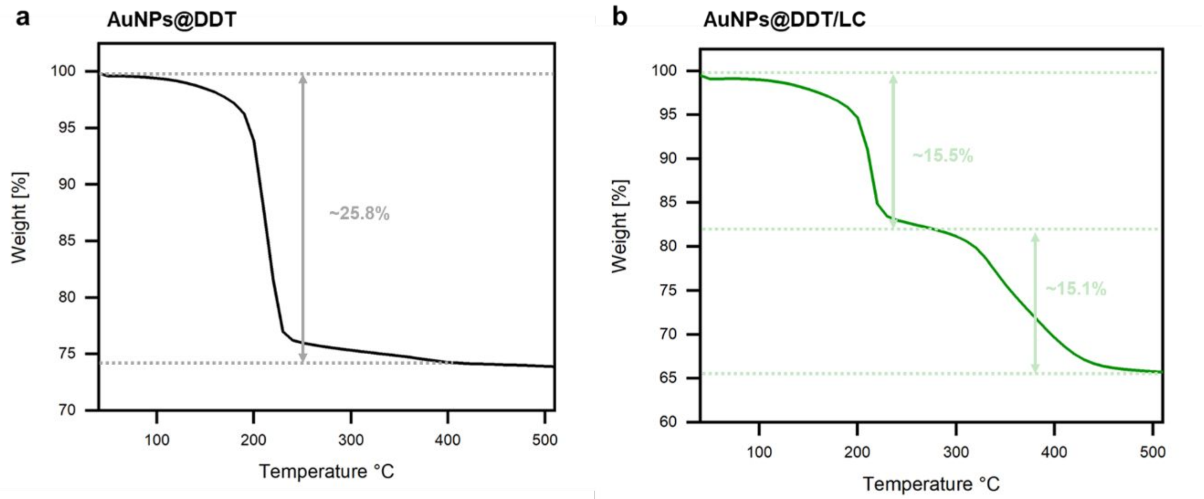

Figure S14. TGA trace of (a) AuNPs@DDT and (b) AuNPs@DDT/LC.

Supporting Note 6. Absorbance measurements of nanoparticles.

Dispersions of AuNPs@DDT and AuNPs@DDT/LC show overlapping plasmon bands at ~495 nm, confirming that ligand exchange does not induce aggregation. Dropcasted NPs films red-shift and broaden to ~525 nm (DDT) and ~518 nm (DDT/LC), as expected for a higher effective refractive index and reduced particle separations in the solid. In contrast, the 50% BPLC composite film exhibits a blue-shifted, narrower band at ~504 nm, which is much closer to the dispersion values, indicating reduced plasmon coupling and improved nanoparticle dispersion within the BPLC matrix. This is consistent with reduced plasmonic coupling and a larger average NP–NP separation within the BPLC matrix. No low-energy shoulders or additional bands typical of sintered/aggregated Au nanoparticles are observed.

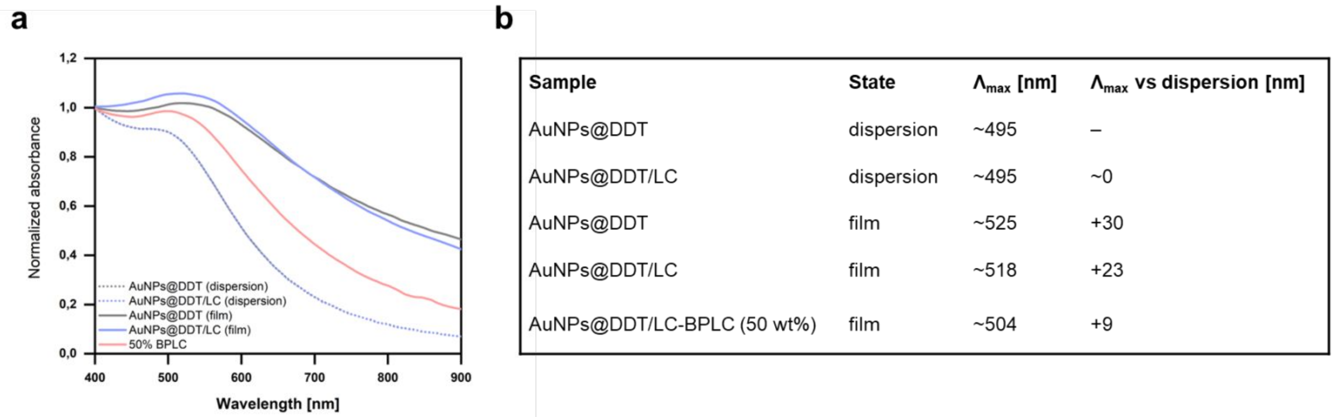

b

Sample	State	Λ_{max} [nm]	Λ_{max} vs dispersion [nm]
AuNPs@DDT	dispersion	~495	–
AuNPs@DDT/LC	dispersion	~495	~0
AuNPs@DDT	film	~525	+30
AuNPs@DDT/LC	film	~518	+23
AuNPs@DDT/LC-BPLC (50 wt%)	film	~504	+9

Figure S15. (a) Normalized UV-Vis spectra of AuNPs@DDT and AuNPs@DDT/LC recorded for dispersions (dotted; grey and blue, respectively) and for drop-cast thin films (solid; grey and blue). The spectrum of a 50 wt% AuNPs@DDT/LC–BPLC composite film is shown in red. (b) Summary of the LSPR bands positions for recorded UV-Vis spectra.

Supporting Note 7. X-ray measurements of nanoparticles.

SAXS measurements probe the spatial organization of the Au cores. Since the interparticle periodicities are imposed by the organic corona, these data can be used to infer the effective shape and rearrangement of the ligand shell. At 30 °C, AuNPs@DDT display a single correlation peak corresponding to a real-space periodicity of ~3.3 nm, i.e., a center-to-center distance consistent with a ~2.2–2.3 nm Au core surrounded by a compact alkyl shell. In contrast, AuNPs@DDT/LC at 30 °C exhibit two well-defined peaks corresponding to periodicities of ~6.9 and 3.4 nm. The shorter distance closely matches the AuNPs@DDT spacing, indicating that within the layers, the in-plane interparticle distance is still primarily defined by the short DDT ligands and the close packing of the Au cores. The longer periodicity (~6.9 nm) reflects an additional structural motif: a layered (lamellar) arrangement with increased separation between successive NP layers, consistent with the presence of longer LC-like ligands extending above and below the cores.

Upon heating AuNPs@DDT/LC to 140 °C, the two peaks merge and shift toward higher angles ($d \approx 4.4$ and 3.5 nm), which we attribute to softening/melting of the mixed organic shell and the loss of long-range lamellar ordering, leaving a more isotropic, short-range hexagonal arrangement of NP cores. On cooling back to 30 °C, the peak positions return close to their original values, confirming that this structural rearrangement is reversible and not associated with aggregation or sintering. Taken together, these observations suggest that at low temperature, the longer LC-like ligands tend to preferentially occupy positions above and below the Au cores, effectively "thickening" the interlayer spacing and giving the NP plus corona an anisotropic (ellipsoidal/cylindrical) effective shape, while the shorter DDT ligands mainly dictate the in-plane spacing. At elevated temperature, this anisotropy is lost as the mixed shell behaves as a molten, more isotropic layer.

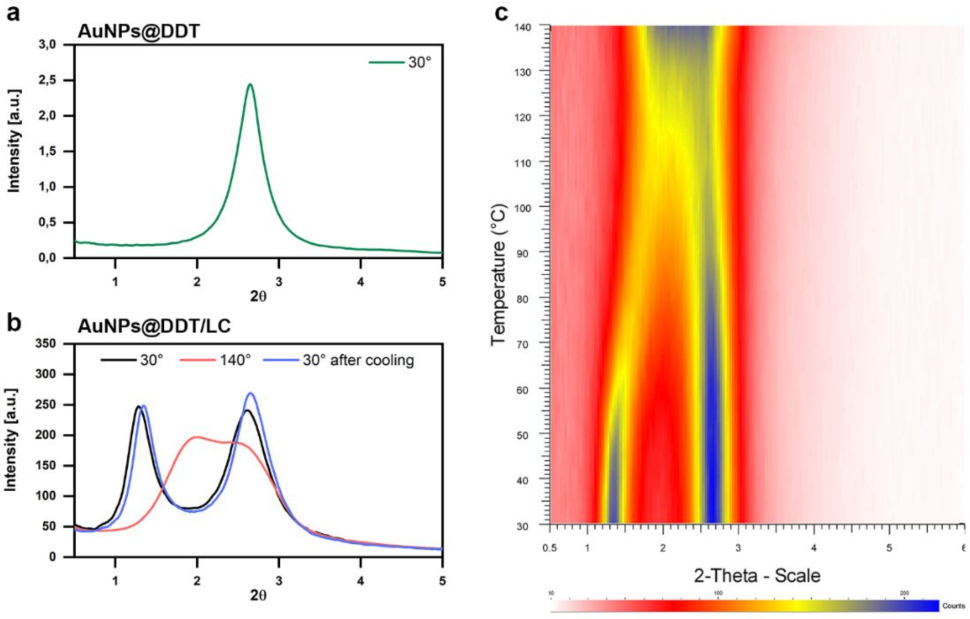

Figure S16. SAXS measurements of AuNPs@DDT and AuNPs@DDT/LC samples. (a) SAXS profile of AuNPs@DDT (before ligand exchange) at 30 °C, showing a single correlation peak at 2θ = 2.65° (periodicity d ≈ 3.33 nm), consistent with a reasonable center-to-center spacing for ~2.3 nm cores coated with C12 ligands. (b) SAXS profiles of AuNPs@DDT/LC at selected temperatures: 30 °C (before heating), 140 °C, and 30 °C after cooling. Peak positions evolve from 2θ = 1.28° and 2.61° (d ≈ 6.9 nm and 3.38 nm) at 30 °C to 2.00° and 2.50° (4.41 nm and 3.53 nm) at 140 °C, and revert to 1.35° and 2.65° (6.54 nm and 3.33 nm) after cooling. (c) Temperature map for AuNPs@DDT/LC, collected every 5 °C from 30 → 140 °C (vertical axis: temperature; horizontal axis: 2θ). The correlation maxima shift with temperature and return on cooling, evidencing thermally driven, reversible rearrangement of the NP superlattice. The absence of a low-angle intensity upturn indicates no macro-aggregation;

the behavior is consistent with a flexible organic corona that compresses/relaxes with T.

Supporting Note 8. Photoinduced birefringence measurements of the PI-A material.

Photoinduced birefringence (Δn) measurements after the PI-A sample irradiation with 445 nm light were performed using a 690 nm probe beam from a diode laser. The measurement technique was based on detecting the transmission (T) of the probe beam through the sample placed between two crossed polarizers[12]. To maximize the transmission signal, the sample was rotated in the vertical plane to a position where its optical axis formed a 45-degree angle with the axes of the polarizers. The value of Δn was calculated using the formula: $\Delta n = \lambda \cdot (\pi d)^{-1} \cdot \arcsin(T^{1/2})$ [12], where λ is the probe beam wavelength and d is the film thickness, measured with a Dektak XT stylus profiler. The obtained birefringence values Δn were 0.02 and 0.04 for samples exposed to light intensities of 80 and 150 mW·cm^{-2}, respectively.

Supporting Tables

Table S1. Composition of the nematic mixture.

No.	Formula	Concentration in weight (%)
1	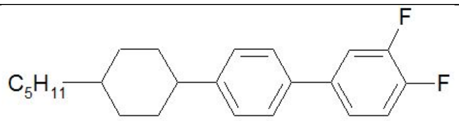	19.8
2	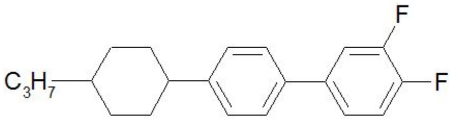	7.2
3	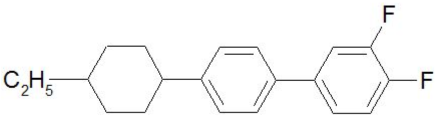	6.5
4	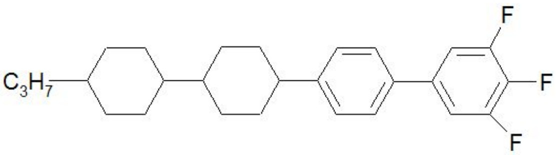	10.8

5	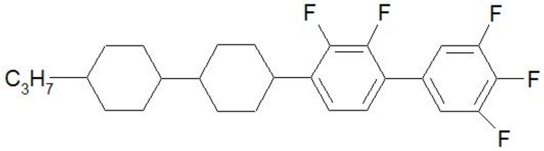	2.5
6	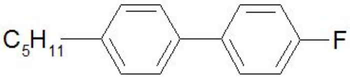	12.5
7	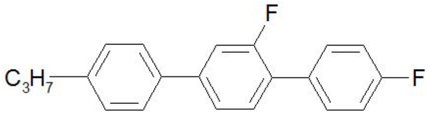	7.1
8	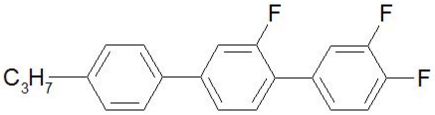	6.1
9	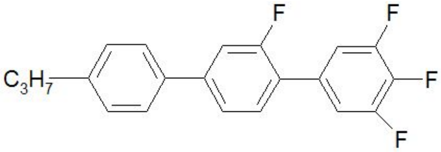	12.9
10	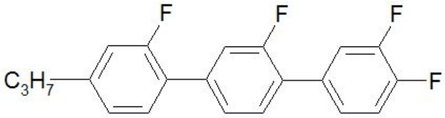	8.4
11	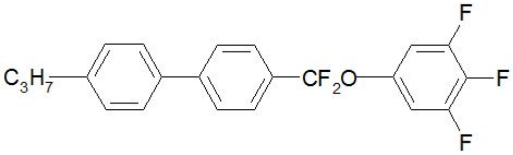	6.2

Table S2. The formula of chiral dopants and their concentrations used in the examined BPLC mixtures.

No.	Formula	*Concentration in the nematic mixture (%)
1	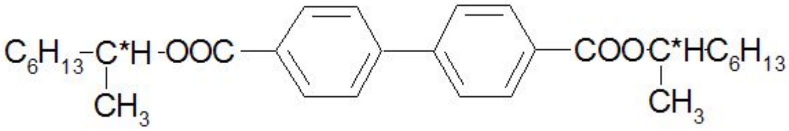	5.7
		7.0
		8.7
2	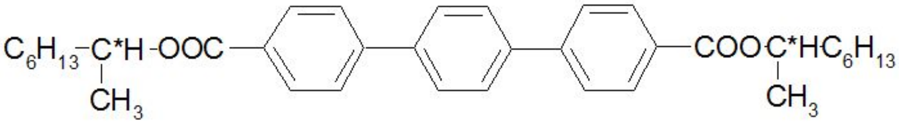	5.4
		7.2
		8.7

*BPLC #1, BPLC #2, BPLC #3

Table S3. Results of Bragg wavelengths and molecular structure analysis of BPLC #2 under the application of gradient temperature.

BP type and cubic unit cell orientation specified by the Miller indices	Bragg wavelength		Lattice constant		Helical pitch	
	Equation*	λ_B (nm)	a (nm)	Δa** (nm)	p (nm)	Δp** (nm)
BPII$_{(100)}$ ($a \sim p/2$)	$\lambda_B = 2\,\tilde{n}\,a = \tilde{n}\,p$	565	166	-	332	-
		590	173	7	346	14
		610	179	6	358	12
BPI$_{(110)}$ ($a \sim p$)	$\lambda_B = \sqrt{2}\,\tilde{n}\,a = \sqrt{2}\,\tilde{n}\,p$	660	278	26	278	26
		720	304		304	

*The average refractive index ($\tilde{n}$) of BPLC in BPII and BPI, based on our previous studies, is 1.7024 and 1.6773, respectively
**Δa and Δp represent the relative change between the successive values of the lattice constant and the helical pitch within a single BP type, respectively

Table S4. Chemical materials to homogeneously align the LC used in the BPLC study.

No.	Name with abbreviation	Alignment technique	Formula	Azimuthal surface anchoring energy ($\times 10^{-6}$ J·m^{-2})

1	Brilliant yellow, BY	photoalignment	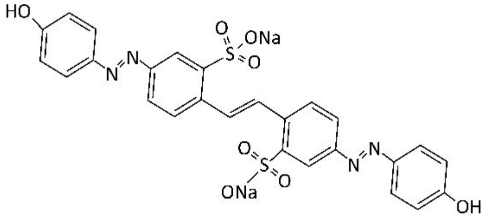	0.1 – 2.2[5]
2	Polyvinyl alcohol, PVA	rubbing	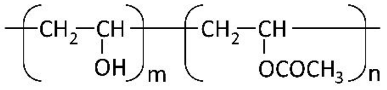	10 – 160[6,7]
3	Azo poly(amide imide), PI-A	photoalignment	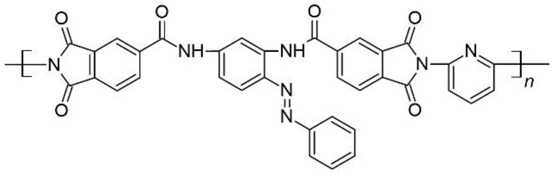	22[8]
4	Polyimide, SE130*	rubbing	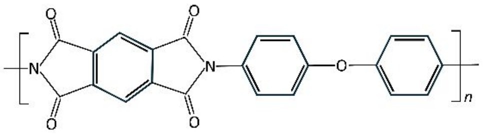	>1000[7,9]

*A chemical formula is presented here for general polyimides; the formula for SE130 is proprietary to Nissan Chemical Industries, Ltd. company.

Table S5. Contact angle measurement procedure and results for all tested ALs; the values reported in this table correspond to measurements obtained using deionized water.

Procedure for the contact angle measurements		
Step 1<br>Water dispensing from a syringe needle	Step 2<br>The substrate rises to make contact with the water droplet	Step 3<br>Contact angle measurement at the water–substrate interface

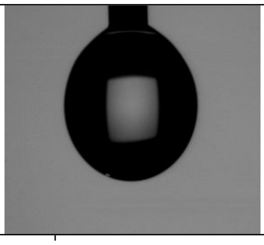

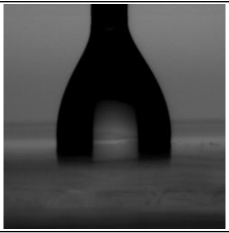

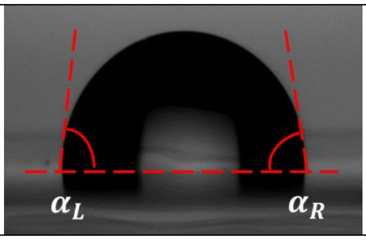

No.	Contact angle measurements							
	BY		PVA		PI-A		SE130	
	α_L (°)	α_R (°)	α_L (°)	α_R (°)	α_L (°)	α_R (°)	α_L (°)	α_R (°)
1	5.3	5.3	40.8	40.8	68.4	68.4	87.7	87.7
2	5.4	5.4	40.7	40.7	68.3	68.3	87.8	87.8
3	5.5	5.5	40.5	40.5	68.2	68.2	87.7	87.7
4	5.4	5.4	40.4	40.4	68.1	68.1	87.6	87.6
5	5.3	5.3	40.3	40.3	68.1	68.1	87.5	87.5
6	5.4	5.4	40.2	40.2	68.4	68.4	87.6	87.6
7	5.4	5.4	40.0	40.0	68.0	68.0	87.4	87.4
8	5.4	5.4	40.0	40.0	68.0	68.0	87.5	87.5
9	5.4	5.4	39.8	39.8	68.0	68.0	87.5	87.5
10	5.3	5.3	39.7	39.7	67.9	67.9	87.3	87.3
*$\bar{\alpha}$	**5.4**		**40.2**		**68.1**		**87.6**	
**σ	**0.1**		**0.3**		**0.2**		**0.1**	

*$\bar{\alpha} = \frac{1}{n}\sum_{i=1}^{n=20} \alpha_i$

**$\sigma = \sqrt{\frac{1}{n}\sum_{i=1}^{n=20} (\alpha_i - \bar{\alpha})^2}$

Table S6. Contact angle measurements for the 1912 nematic LC and BPLC samples, including tested ALs at different temperatures.

Temperature (°C)	*α (°)			
	PI-A		SE130	
	nematic	BPLC	nematic	BPLC
25	20.1	19.5	30.0	30.9
45	-	19.2	-	29.8
50	18.9	19.1	29.9	28.8
55	-	19.1	-	28.4
60	18.6	19.0	29.8	28.0
70	18.4	19.0	29.4	27.6

*The results for BY and PVA ALs were approximately 0°

Table S7. Positions of the peaks in the SAXS profiles of AuNPs@DDT/LC-BPLC composites.

Sample	Temperature (°C)	2ϑ (°)	Periodicity (nm)
AuNPs@DDT/LC	30	1.28; 2.61	6.90; 3.38
AuNPs@DDT/LC	140	2.00; 2.50	4.41; 3.53
AuNPs@DDT/LC–BPLC (5 wt%)	30	1.23; 2.46	7.17; 3.58
AuNPs@DDT/LC–BPLC (5 wt%)	140	1.89	4.67
AuNPs@DDT/LC–BPLC (50 wt%)	30	1.30; 2.60	6.79; 3.39
AuNPs@DDT/LC–BPLC (50 wt%)	140	1.73	5.10

Table S8. Thermal and photonic characteristics of the BPLC #2 composites in cells with various types of homogeneous ALs.

Type of AL	NP concentration in BPLC (wt%)	Type of BP	Temperature range		Bragg wavelength (nm)
			T (°C)	ΔT (°C)	
BY	0	BPI	52.2 – 56.4	4.2	756
		BPII	56.4 – 58.2	1.8	627
	0.5	BPI	50.4 – 56.0	5.6	735
		BPII	56.0 – 58.2	2.2	600
	2.0	BPI	50.7 – 56.1	5.4	720
		BPII	56.1 – 58.6	2.5	590
PVA	0	BPI	52.4 – 56.4	4.0	755
		BPII	56.4 – 58.0	1.6	620
	0.5	BPI	51.6 – 56.8	5.2	730
		BPII	56.8 – 58.0	1.2	600
	2.0	BPI	51.0 – 57.2	6.2	714
		BPII	57.2 – 59.0	1.8	583
PI-A	0	BPI	53.3 – 57.7	4.4	721
		BPII	57.7 – 60.0	2.3	607
	0.5	BPI	44.2 – 55.9	11.7	690
		BPII	55.9 – 57.8	1.9	572
	2.0	BPI	43.8 – 58.0	14.2	659
		BPII	58.0 – 59.3	1.3	565
SE130	0	BPI	54.5 – 58.0	3.5	717
		BPII	58.0 – 60.0	2.0	595
	0.5	BPI	55.0 – 59.5	4.5	669
		BPII	59.5 – 61.7	2.2	561
	2.0	BPI	45.4 – 61.2	15.8	637
		BPII	61.2 – 63.8	2.6	557

Table S9. Comparison of the lattice constants calculated using the Bragg wavelength equation (a_{calc}) and simulated based on fitting lines to the Kossel patterns (a_{sim}) for $BPI_{(110)}$.

Type of AL	NP concentration in BPLC (wt%)	Bragg wavelength (nm)	Lattice constant	
			a_{calc} (nm)	a_{sim} (nm)
BY	0	756	318	318
	0.5	735	307	313
	2.0	720	302	310
PVA	0	755	318	318
	0.5	730	305	308
	2.0	714	295	301
PI-A	0	721	304	304
	0.5	690	288	296
	2.0	659	272	284
SE130	0	717	302	302
	0.5	669	279	279
	2.0	637	263	263

Table S10. Average nanoparticle size and size dispersion before ligand exchange, after ligand exchange, and after dispersion in BPLC mixtures.

Sample	NP size (nm)
AuNPs@DDT	2.3 ± 0.4
AuNPs@DDT/LC	2.2 ± 0.4
AuNP@DDT/LC – BPLC (5 wt%)	2.2 ± 0.3
AuNPs@DDT/LC – BPLC (50 wt%)	2.2 ± 0.4

Supporting References:

(1) Zink, H.; Belyakov, V. A. Temperature Variations in the Director Orientation and Anchoring Energy at the Surface of Cholesteric Layers. *J. Exp. Theor. Phys. Lett.* **1996**, *63* (1), 43–49. https://doi.org/10.1134/1.566961.

(2) Gandhi, J. V.; Mi, X.-D.; Yang, D.-K. Effect of Surface Alignment Layers on the Configurational Transitions in Cholesteric Liquid Crystals. *Phys. Rev. E* **1998**, *57* (6), 6761–6766. https://doi.org/10.1103/PhysRevE.57.6761.

(3) Yoon, H. G.; Roberts, N. W.; Gleeson, H. F. An Experimental Investigation of Discrete Changes in Pitch in a Thin, Planar Chiral Nematic Device. *Liq. Cryst.* **2006**, *33* (4), 503–510. https://doi.org/10.1080/02678290600633501.

(4) McKay, G. Bistable Surface Anchoring and Hysteresis of Pitch Jumps in a Planar Cholesteric Liquid Crystal. *Eur. Phys. J. E* **2012**, *35* (8), 74. https://doi.org/10.1140/epje/i2012-12074-1.

(5) Padmini, H. N.; Rajabi, M.; Shiyanovskii, S. V.; Lavrentovich, O. D. Azimuthal Anchoring Strength in Photopatterned Alignment of a Nematic. *Crystals* **2021**, *11* (6), 675. https://doi.org/10.3390/cryst11060675.

(6) Cui, Y.; Zola, R. S.; Yang, Y.-C.; Yang, D.-K. Alignment Layers with Variable Anchoring Strengths from Polyvinyl Alcohol. *J. Appl. Phys.* **2012**, *111* (6), 063520. https://doi.org/10.1063/1.3697680.

(7) Ishihara, S.; Mizusaki, M. Alignment Control Technology of Liquid Crystal Molecules. *J. Soc. Inf. Disp.* **2020**, *28* (1), 44–74. https://doi.org/10.1002/jsid.825.

(8) Kozanecka-Szmigiel, A.; Rutkowska, K. A.; Nieborek, M.; Kwasny, M.; Karpierz, M. A.; Schab-Balcerzak, E.; Konieczkowska, J.; Szmigiel, D. Photopatterned Azo Poly(Amide Imide) Layers as Aligning Substrates of Holographic Liquid Crystal Diffraction Gratings for Beam Steering Applications. *J. Mater. Chem. C* **2020**, *8* (3), 968–976. https://doi.org/10.1039/C9TC04296B.

(9) Rabilloud, G. High-Performance Polymers: Chemistry and Applications. 3, Polyimides in Electronics. *Inst. Fr. Pétrole Publ.* **2000**, *3*.

(10) Chibowski, E.; Ontiveros-Ortega, A.; Perea-Carpio, R. On the Interpretation of Contact Angle Hysteresis. *J. Adhes. Sci. Technol.* **2002**, *16* (10), 1367–1404. https://doi.org/10.1163/156856102320252859.

(11) Muravsky, A.; Murauski, A. Q&A of Liquid Crystal Alignment: Theory and Practice. *Front. Soft Matter* **2024**, *Volume 4-2024*. https://doi.org/10.3389/frsfm.2024.1382925.

(12) Born, M.; Wolf, E. *Principles of Optics: Electromagnetic Theory of Propagation, Interference and Diffraction of Light*, 7th ed.; Cambridge University Press: Cambridge, 1999. https://doi.org/10.1017/CBO9781139644181.
